# Physical Activity and Cancer Status Among Middle-Aged and Older Chinese: A Population-Based, Cross-Sectional Study

**DOI:** 10.3389/fphys.2021.812290

**Published:** 2022-01-27

**Authors:** Chunsu Zhu, Zhiwei Lian, Ying Chen, Jianmin Wang

**Affiliations:** Fujian Medical University Cancer Hospital, Fujian Cancer Hospital, Fuzhou, China

**Keywords:** physical activity, cancer, epidemiology, cancer survivors, cross-sectional study

## Abstract

**Background:**

The relative contributions of demographic and lifestyle behaviors to the association between physical activity (PA) and cancer are poorly understood. This study assesses the relationship between PA level and cancer status considering the full activity spectrum within a large and representative Chinese population.

**Methods:**

Data were derived from the Chinese Health and Retirement Longitudinal Study (using four-stage stratified probability-proportional-to-size sampling), including 416 cancer survivors and 14,574 individuals without cancer from 28 provinces in China. Cancer status and sites were self-reported, and PA, other health behaviors (e.g., smoking, drinking) and comorbidities (e.g., hypertension, diabetes) were assessed by a questionnaire. The total PA score was calculated using metabolic equivalent (MET) multipliers. Multivariable logistic regression was used to estimate differences in PA levels between cancer survivors and those without a cancer diagnosis, adjusting for age, sex, and other potential confounding factors.

**Results:**

Cancer survivors (416, 2.8%) were more likely to be women than men (65.4 vs. 34.6%). They were older (age ≥65 years, 43.8 vs. 38.9%) and more likely to be overweight (18.3 vs. 13.3%), be depressed (49.5 vs. 37.6%), have quit smoking (17.8 vs. 14.4%), drink less (17.5 vs. 26.6%), sleep less (65.9 vs. 56.8%) and have more chronic comorbidities (≥2 comorbidities, 26.0 vs. 19.2%) than those without cancer. There was a significant associations between cancer status and participation in vigorous-intensity activity for at least 10 min every week, when compared with the inactivity [odds ratio (OR) = 0.56, 95% CI = 0.39–0.80], while no differences were observed in the moderate and light activity groups. Individuals who spent more than half an hour performing moderate or vigorous intensity activity every day were significantly less likely to report a cancer diagnosis than inactive individuals (moderate OR = 0.64, 95% CI = 0.48–0.86; vigorous OR = 0.50, 95% CI = 0.37–0.68). Participants who spent more than 2 h performing light, moderate or vigorous intensity activity reported fewer cancer cases than their inactive counterparts. In addition, there was an inverse dose-response relationship between the total PA score and cancer status (*P_–trend_* < 0.001).

**Conclusion:**

Associations between PA and cancer status were independent of demographics, lifestyle confounders, and comorbidities. Cancer survivors are less physically active than those without cancer.

## Introduction

Cancer is a leading cause of morbidity and mortality around the world and contributes to one in eight deaths globally (Fitzmaurice et al., 2019). In 2017, there were 24.5 million cancer cases and 9.6 million cancer deaths (Fitzmaurice et al., 2019). New cancer incidence is expected to increase by nearly 70% by 2030 (Fitzmaurice et al., 2019). In China, approximately 4.3 million people were diagnosed with cancer in 2015 (Chen et al., 2016), and the prevalence of this disease is growing rapidly due to the aging population and westernized lifestyle (de Magalhães, 2013). Cancer patients can expect to live for decades thanks to earlier detection, better diagnostic and staging methods, and more effective treatments. For example, during the past decade, the number of cancer survivors in China has increased remarkably, and nearly 40.5% of cancer survivors live for more than 5 years (Zeng et al., 2018). Compared with individuals without cancer, cancer survivors are at increased risk for other chronic diseases, secondary complications, recurrence, and decreased physical function and quality of life (Aziz and Rowland, 2003; Jemal et al., 2007). Thus, attenuating secondary health problems in cancer survivors has become a major public health challenge. However, cancer incidence and recurrence can be prevented with a healthy lifestyle, including regular physical activity (PA), to some extent (Loprinzi et al., 2012; Lahart et al., 2015; Murray et al., 2020). PA is a modifiable behavior that is linked to several cancers and has been shown to be effective in the primary prevention of cancer (Moore et al., 2012; Hojman et al., 2018). Efforts to address modifiable risk factors can provide support for economical options to control cancer.

Previous studies have shown that PA has beneficial effects on the risk of developing certain types of cancer and also has favorable influences on outcomes among cancer survivors (Moore et al., 2016; Kerr et al., 2017; Matthews et al., 2020). There is convincing evidence that PA is associated with a lower risk of death and recurrence in cancer survivors, along with psychosocial wellness and life satisfaction (Lahart et al., 2015; Pudkasam et al., 2018; Van Blarigan et al., 2018). Increasing the level of PA participation can improve body composition, cardiopulmonary function, muscle strength and quality of life in cancer survivors, and it can also reduce the rate of cancer recurrence and cancer-related mortality (Haydon et al., 2006; Meyerhardt et al., 2006; Schmid and Leitzmann, 2014). It is essential that we better understand the prevalence and patterns of PA to promote healthy and proper PA among cancer survivors. The second American College of Sports Medicine Roundtable recommends that cancer survivors avoid inactivity and engage in specific levels of PA to improve common cancer-related outcomes, such as anxiety, depressive symptoms, and fatigue (Campbell et al., 2019). Unfortunately, however, few meet or exceed the PA guidelines for cancer survivors (Mo et al., 2021). Individuals with cancer may be less likely to engage in PA than cancer-free participants as a result of their diagnosis and treatment. However, previous studies on PA levels among cancer survivors and cancer-free controls have shown inconsistent results (Park et al., 2015; Wang et al., 2015; Friis et al., 2018; Morris et al., 2018). For instance, some reported that cancer survivors were more likely to have higher PA levels (Park et al., 2015), while others reported that cancer survivors are either not different from or have lower levels of PA than their cancer-free counterparts (Wang et al., 2015; Mowls et al., 2016; Friis et al., 2018). Although very informative, these mixed studies did not assess the wide range of covariates that we were able to include in our study, and some of these previous studies had small sample sizes, which led to limited generalizability. Moreover, the majority of these studies were carried out in Western populations. To the best of our knowledge, no large-scale population-based study in China to date has assessed the associations between PA level and cancer status while accounting for a wide range of potential confounding factors. Comparisons and assessments of the association between PA level and cancer status may inform the development and implementation of evidence-based PA recommendations for these survivors.

Therefore, the purpose of this study was threefold. First, we wanted to compare PA levels between cancer patients and their cancer-free counterparts while considering a wide range of cofounders within a large, nationally representative Chinese population aged ≥45 years. Second, we wanted to examine the potential dose-response relationship between the total PA score and cancer status. Finally, we wanted to explore whether demographic characteristics, unhealthy lifestyles and chronic comorbidities influenced these associations.

## Materials and Methods

### Participants

The data used in this study were derived from the fourth wave of surveys of the China Health and Longitudinal Study (CHARLS), which is a nationally representative longitudinal survey of household residents aged ≥45 years in mainland China, with 19,752 individuals from 28 provinces, 150 countries/districts, and 450 villages/urban communities surveyed from March, 2018 to March, 2019. In the current study, we excluded those with missing values for cancer status (*n* = 47), PA (*n* = 339), age (*n* = 76), body mass index (BMI) (*n* = 2901), depressive symptoms (*n* = 1950), residence (*n* = 1603), and smoking (*n* = 3). Finally, 14,990 adults were included in our analysis. No analysis plan was prespecified or registered for this study.

### Physical Activity

A modified version of the International Physical Activity Questionnaire-Short Form (IPAQ-SF) was used to measure PA in terms of vigorous, moderate, and light activity (see Supplementary Material; Craig et al., 2003). The participants were asked about the amount of time they spent on different types of PA during a typical week. Vigorous activity was defined as activities requiring hard/high-intensity physical effort and making breath much harder than normal, such as heavy lifting, digging, plowing, aerobics, fast bicycling, and cycling with a heavy load. Moderate activity refers to activities that take moderate physical effort and make breath somewhat harder than normal and may include carrying light loads, bicycling at a regular pace, or mopping the floor. Light activity was defined as walking at work and at home, traveling from place to place and any other walking for recreation, sport, exercise or leisure. The response to the amount of PA in 1 day was indexed as 1 ≤ 0.5 h; 2 = 0.5–2 h; 3 = 2–4 h; and 4 ≥ 4 h (Deng and Paul, 2018). The PA duration score for a week was calculated by multiplying the daily PA duration index for each activity by the number of days. Then, the total PA score was calculated with metabolic equivalent (MET) multipliers as follows: total PA score = 8 × total vigorous activity weekly duration score + 4.0 × total moderate activity weekly duration score + 3.3 × total light activity weekly duration score (Deng and Paul, 2018). The total PA score was classified into four groups according to the 25th percentile, median and 75th percentile: Q1 = <Q_25_, Q2 = Q_25_–Q_50_, Q3 = Q_50_–Q_75_, and Q4 = ≥Q_75_.

### Cancer

Participants were asked the question “Have you been diagnosed with cancer or a malignant tumor (excluding minor skin cancers) by a doctor?” Individuals who reported having a cancer history were further asked about the site or organ of cancer. The sensitivity for self-reported cancer varied from 0.79 to 0.93, using the registry reports of cancer as the standard (Bergmann et al., 1998).

### Covariates

Demographic characteristics were self-reported, including age (used as a continuous variable), sex (male, female), marital status (married, separated/divorced/widowed/never married), education level (below high school, high school, and above), and place of residence (rural, urban). Health-related behaviors included smoking (never smoked, quit smoking, current smoker), drinking frequency in the past (never, ≤1/month, >1/month), and sleep duration (7–8 h, <7 h, ≥8 h). Sleep duration was assessed with the question “During the past month, how many hours of actual sleep did you get at night?” BMI was calculated as the weight in kilograms divided by the square of height in meters, and obesity was defined as BMI ≥ 28.0 kg/m^2^ according to the guidelines for Chinese people. Depressive symptoms (yes/no) were measured using the 10-item version of the Center for Epidemiological Studied Depression Scale (CESD-10), and a total score of at least 10 was classified as clinically elevated depressive symptoms (Chen and Mui, 2014). In this study, chronic physical comorbidities included hypertension, diabetes, heart problems (including heart attack, coronary heart disease, angina, congestive heart failure, or other heart problems), chronic lung diseases (including chronic bronchitis, emphysema, and excluding tumors or cancers), liver diseases (except fatty liver, tumors, or cancers), stroke, kidney disease (except for tumor or cancers), stomach or other digestive disease (except for tumor), and arthritis or rheumatism. Hypertension was defined based on a history of hypertension, intake of antihypertensive drugs, and measurement of blood pressure (systolic blood pressure ≥140 mmHg or diastolic blood pressure ≥90 mmHg). Diabetes was diagnosed based on self-reported medical history and blood measurements. The other chronic diseases were defined by a self-reported doctor diagnosis. Physical comorbidities were categorized as 0, 1, or ≥2 according to the number of chronic diseases.

### Statistical Analysis

Descriptive statistics of participant characteristics are presented according to the self-reported doctor diagnosis of cancer (yes/no), continuous variables are presented as the means and SD, and categorical variables are shown as the counts and frequency. To compare the characteristics of cancer survivors and participants without cancer, Student’s *t* test was adopted for normally distributed continuous variables, and the chi squared test was adopted for categorical variables. To examine whether there were differences in PA between cancer survivors and cancer-free participants, three binary logistic regression models were fitted with cancer status as the dependent variable and PA as the independent variable. Odds ratios (ORs) and 95% confidence intervals (CIs) were calculated to show the association between cancer and PA. Model 1 was unadjusted to calculate the crude OR and 95% CI. Model 2 was adjusted for demographic characteristics (including age, sex, marital status, education, and place of residence). Model 3 was further adjusted for health-related behaviors (smoking, drinking frequency, sleep duration, and obesity), depressive symptoms and chronic comorbidities (Shi et al., 2017; Cortés-Ibáñez et al., 2020). We retained the covariates, even if they were not statistically significant in changing the association between PA and cancer, to see if PA was independent of these variables. To assess the linear trends between PA and cancer status, Wald tests were performed, considering the total PA score categories as a continuous variable. To assess whether the relationship between being a cancer patient and PA was more prominent in specific subgroups, stratification analyses by all aforementioned variables were carried out, and the chi square-based *Q* test was used to test the heterogeneity. Interaction analyses were further performed by entering the interaction terms into the logistic regression model. In the sensitivity analyses, missing value analyses were conducted to identify the missingness mechanism of missing variables; based on these results, we hypothesized that the data used in this study were missing at random (Siddique et al., 2008). Therefore, we excluded individuals with missing values less than 10%, variables with more than 10% missing data were imputed with the median or mean (this was the case for BMI), and logistic regression analyses were repeated. R software was used to draw figures, as well as perform subgroup and interaction analyses. The relevant packages and codes are provided in the Supplementary Material. Other analyses were conducted using SPSS version 21.0, two-sided *P*-values < 0.05 were considered to be statistically significant for the main analyses. To reduce the false discovery rate, two-sides *P*-value < 0.01 was used for subgroup analyses.

## Results

### Participants’ Characteristics

The characteristics of the participants are presented in Table 1. A total of 14,990 individuals were included, with 416 (2.8%) reporting a history of cancer. Among these cancer survivors, 12.5% had breast cancer, 7.2% had lung cancer, 15.4% had cervix cancer, 5.5% had liver cancer, 7.0% had endometrium cancer, 6.3% had colon or rectum cancer, 6.3% had stomach cancer, 4.1% had thyroid cancer, 2.9% had brain cancer, 3.4% had esophagus cancer, 2.4% had kidney cancer, 2.6% had ovary cancer, 18.5% had cancer of another organ, 6.0% had cancer of an unknown site. The cancer survivors were older than those without cancer (mean age 63.3 ± 9.1 vs. 61.9 ± 9.6 years), and were more likely to be women (*p* < 0.001), live in an urban area (*p* = 0.006), be obese (*p* = 0.003), quit smoking (*p* < 0.001), drink less (*p* < 0.001), sleep less (*p* < 0.001), have depressive symptoms (*p* < 0.001), have physical comorbidities (*p* < 0.001), and be physically inactive (*p* < 0.001) (Table 1 and Supplementary Table 1).

**TABLE 1:** Characteristics of study participants in the fourth wave of CHARLS.

Characteristic	Total (*N* = 14,990)	Cancer survivors (*N* = 416)	Non-cancer (*N* = 14,574)	*P*-value
Age, year, mean (SD)	61.9 (9.6)	63.3 (9.1)	61.9 (9.6)	0.045
Sex, n (%)				<0.001
Male	7015 (46.8)	144 (34.6)	6871 (47.1)	
Female	7975 (53.2)	272 (65.4)	7703 (52.9)	
Education, n (%)				0.690
Below high school	13245 (88.4)	365 (87.7)	12880 (88.4)	
High school and above	1745 (11.6)	51 (12.1)	1694 (11.6)	
Marital status, n (%)				0.404
Married	12928 (86.2)	353 (84.9)	12575 (86.3)	
Separated/divorce/ widowed/never married	2062 (13.8)	63 (15.1)	1999 (13.7)	
Place of residency, n (%)				0.006
Rural	11208 (74.8)	287 (69.0)	10921 (74.9)	
Urban	3782 (25.2)	129 (31.0)	3653 (25.1)	
BMI, kg/m_2_, n (%)				0.003
<27.9 (not obesity)	12976 (86.6)	340 (81.7)	12636 (86.7)	
≥28.0 (obesity)	2014 (13.4)	76 (18.3)	1938 (13.3)	
Smoking status, n (%)				<0.001
Never	8780 (58.6)	274 (65.9)	8506 (58.4)	
Quit	2170 (14.5)	74 (17.8)	2096 (14.4)	
Current smoker	4040 (27.0)	68 (16.3)	3972 (27.3)	
Drinking frequency, n (%)				<0.001
Never	9921 (66.2)	307 (73.8)	9614 (66.0)	
Less than once a month	1114 (7.4)	36 (8.7)	1078 (7.4)	
More than once a month	3955 (26.4)	73 (17.5)	3882 (26.6)	
Sleep duration, hours, n (%)				<0.001
7–8	2495 (16.6)	69 (16.6)	2426 (16.6)	
<7	8548 (57.0)	274 (65.9)	8274 (56.8)	
≥8	3947 (26.3)	73 (17.5)	3874 (26.6)	
Depressive symptoms, n (%)				<0.001
No	9311 (62.1)	210 (50.5)	9101 (62.4)	
Yes	5679 (37.9)	206 (49.5)	5473 (37.6)	
Comorbidities, n (%)				<0.001
0	7176 (47.9)	151 (36.3)	7025 (48.2)	
1	4904 (32.7)	157 (37.7)	4747 (32.6)	
≥2	2910 (19.4)	108 (26.0)	2802 (19.2)	
Cancer site, n (%)				
Brain	12 (0.08)	12 (2.9)		
Lung	30 (0.20)	30 (7.2)		
Thyroid	17 (0.11)	17 (4.1)		
Breast	52 (0.35)	52 (12.5)		
Esophagus	14 (0.09)	14 (3.4)		
Stomach	26 (0.17)	26 (6.3)		
Liver	23 (0.15)	23 (5.5)		
Kidney	10 (0.07)	10 (2.4)		
Ovary	11 (0.07)	11 (2.6)		
Cervix	64 (0.43)	64 (15.4)		
Endometrium	29 (0.19)	29 (7.0)		
Colon or rectum	26 (0.17)	26 (6.3)		
Other organs	77 (5.1)	77 (18.5)		
Unknown site	25 (0.17)	25 (6.0)		

*SD, standard deviation. Chi square test and t-test were used as appropriate.*

Compared with cancer-free participants, cancer survivors were more often women than men (OR = 1.63, 95% CI = 1.18–2.24) and were more likely to quit smoking (OR = 1.60, 95% CI = 1.13–2.27), sleep less than 8 h (OR = 0.66, 95% CI = 0.47–0.92), and have depressive symptoms (OR = 1.46, 95% CI = 1.19–1.79) or chronic comorbidities (1 chronic disease: OR = 1.42, 95% CI = 1.13–1.79, ≥2 chronic diseases: OR = 1.41, 95% CI = 1.09–1.84) (Supplementary Table 2).

### Logistic Regression Models to Describe the Association Between Physical Activity and Cancer

Table 2 presents the multivariate logistic regression results for the associations between PA level and cancer. In model 1, there was a significant association between cancer and taking part in vigorous activity for at least 10 min in a usual week, compared with inactivity (OR = 0.48, 95% CI = 0.34–0.68), and no significant differences were observed between groups in terms of moderate or light activity (*p* > 0.05). Participants with cancer diagnoses were less likely to spend more than 30 min (light OR = 0.75, 95% CI = 0.58–0.99; moderate: OR = 0.64, 95% CI = 0.48–0.85; vigorous: OR = 0.43, 95% CI = 0.32–0.58) or 2 h per day (light: OR = 0.71, 95% CI = 0.53–0.94; moderate: OR = 0.58, 95% CI = 0.42–0.80; vigorously: OR = 0.40, 95% CI = 0.30–0.54) on light, moderate and vigorous-intensity PA than the cancer-free participants. In addition, an inverse dose-response relationship was detected between the total PA score and cancer status (*p_–trend_* < 0.001). In model 2, after adjusting for demographic characteristics, the associations between PA patterns and cancer were slightly altered, but the statistical significance remained similar. In model 3, after full adjustment, the associations between PA level and cancer were slightly lower. Individuals with cancer status were less likely to perform vigorous PA for at least 10 min every week than cancer-free individuals (OR = 0.56, 95% CI = 0.39–0.80). Nevertheless, the differences between groups were not significant across light and moderate PA (*p* > 0.05). Adults with cancer status were less likely to participate in light, moderate or vigorous PA for more than 30 min or 2 h. In addition, there was a significant dose-response relationship between the total PA score and cancer (*p_–trend_* < 0.001).

**TABLE 2:** Logistic regression analyses of associations between different physical activity levels and cancer status.

Variables	Total n (%)	Model 1		Model 2		Model 3	
		OR (95% CI)	*P*-value	OR (95% CI)	*P*-value	OR (95% CI)	*P*-value
**Taking part in activity more than 10 min a week**							
Inactive	1337 (8.9)	1		1		1	
Light	5034 (33.6)	0.90 (0.65, 1.25)	0.539	0.92 (0.66, 1.27)	0.613	0.96 (0.69, 1.33)	0.803
Moderate	4311 (28.8)	0.73 (0.53, 1.02)	0.069	0.70 (0.50, 0.99)	0.041	0.75 (0.53, 1.05)	0.091
Vigorous	4308 (28.7)	0.48 (0.34, 0.68)	<0.001	0.53 (0.37, 0.76)	<0.001	0.56 (0.39, 0.80)	0.001
**Time usually spend doing activity ≥30 min 1 day**							
Inactive	2224 (14.8)	1		1		1	
Light	4880 (32.6)	0.75 (0.58, 0.99)	0.040	0.76 (0.58, 0.99)	0.044	0.79 (0.60, 1.04)	0.087
Moderate	3737 (24.9)	0.64 (0.48, 0.85)	0.002	0.61 (0.46, 0.82)	0.001	0.64 (0.48, 0.86)	0.003
Vigorous	4149 (27.7)	0.43 (0.32, 0.58)	<0.001	0.48 (0.35, 0.64)	<0.001	0.50 (0.37, 0.68)	<0.001
**Time usually spend doing activity ≥2 h 1 day**							
Inactive	6576 (43.9)	1		1		1	
Light	3958 (26.4)	0.71 (0.53, 0.94)	0.016	0.71 (0.54, 0.95)	0.019	0.73 (0.55, 0.97)	0.029
Moderate	2134 (14.2)	0.58 (0.42, 0.80)	0.001	0.58 (0.42, 0.80)	0.001	0.60 (0.44, 0.83)	0.002
Vigorous	2322 (15.5)	0.40 (0.30, 0.54)	<0.001	0.45 (0.33, 0.60)	<0.001	0.46 (0.34, 0.62)	<0.001
**Time usually spend doing activity ≥4 h 1 day**							
Inactive	9999 (66.7)	1		1		1	
Light	2797 (18.7)	0.65 (0.42, 1.01)	0.056	0.68 (0.44, 1.05)	0.082	0.70 (0.45, 1.09)	0.112
Moderate	1157 (7.7)	0.75 (0.51, 1.11)	0.145	0.79 (0.53, 1.18)	0.249	0.81 (0.54, 1.20)	0.291
Vigorous	1037 (6.9)	0.49 (0.36, 0.68)	<0.001	0.57 (0.41, 0.78)	0.001	0.58 (0.42, 0.81)	0.001
**Total PA score**							
Q1	2869 (19.1)	1		1		1	
Q2	4626 (30.9)	0.79 (0.62, 1.02)	0.075	0.80 (0.62, 1.03)	0.086	0.83 (0.64, 1.07)	0.142
Q3	3747 (25.0)	0.65 (0.49, 0.85)	0.002	0.64 (0.48, 0.85)	0.002	0.66 (0.50, 0.88)	0.005
Q4	3748 (25.0)	0.48 (0.35, 0.65)	<0.001	0.53 (0.39, 0.73)	<0.001	0.56 (0.41, 0.76)	<0.001
*P-trend*		<0.001		<0.001		<0.001	

*OR, odds ratio; CI, confidence interval. Model 1 was unadjusted; Model 2 was adjusted for age, sex, education, marital status, place of residence; Model 3 was further adjusted for smoking, drinking, obesity, sleep duration, depressive symptoms, and chronic comorbidity.*

### Subgroup Analyses

Stratification analyses for the associations between PA and cancer were then conducted by all aforementioned variables. In Supplementary Material (Supplementary Tables 3–13), the associations between cancer status and PA were more prominent in individuals aged ≥65 years, males, those living in urban areas, those who were married, those with a lower education level, those with BMI < 28.0 kg/m^2^, those who were depressed, former smokers, those whose sleep duration was <7 h, those with chronic comorbidities. However, heterogeneity among the strata was only significant for sleep duration (*p* = 0.006).

**FIGURE 1:**
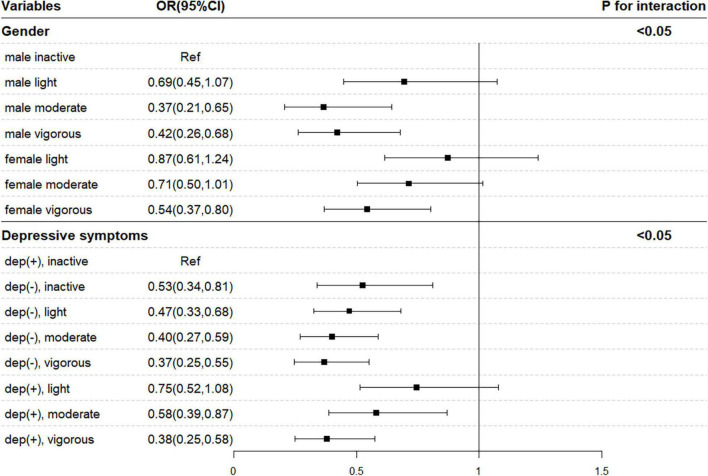
Interaction analysis between sex, depressive symptoms, and physical activity. OR, odds ratio; CI, confidence interval. Model was adjusted for age, sex, education, marital status, place of residence, smoking, drinking, obesity, sleep duration, depressive symptoms, and chronic comorbidities.

Then, statistically significant multiplicative interactions between PA and sex and depressive symptoms were identified (Figure 1, *p*_–interaction_ < 0.05). Females with cancer status were less likely to spend more than 30 min performing vigorous PA every day than males (OR = 0.54, 95% CI = 0.37–0.80), after adjusting for age, marital status, education level, place of residence, BMI, smoking, drinking, sleep duration, depressive symptoms, and physical comorbidities. Compared with cancer-free individuals, those with cancer were more likely to be physically inactive and have depressive symptoms (OR = 0.37, 95% CI = 0.25–0.55).

### Sensitivity Analyses

In sensitivity analyses, after the missing BMI values were replaced with the median, the logistic regression analysis results showed robustness. Individuals who were diagnosed with cancer tended to be more physically inactive than their cancer-free counterparts after full adjustment (OR = 0.54, 95% CI = 0.38–0.76). Those who had cancer diagnoses were less likely to spend more than 30 min or 2 h every day performing PA than cancer-free individuals, regardless of the PA intensity. Moreover, a significant dose-response relationship was observed between the total PA score and cancer status (*p_–trend_* < 0.001) (Supplementary Figure 1).

## Discussion

In this cross-sectional analysis, we compared the PA levels of 14,574 individuals without cancer and 416 cancer survivors from 28 provinces in mainland China. We found that participants with cancer were more likely to be physically inactive than their cancer-free counterparts in both the unadjusted and adjusted analyses. Individuals with cancer diagnoses were less likely to spend more than 10 min performing vigorous activity each week than cancer-free individuals, while no significant differences existed between groups that performed light or moderate activity, independent of demographic characteristics, health-related lifestyle factors, depressive symptoms and chronic comorbidities. Cancer patients had a lower tendency to perform PA for more than 30 min or 2 h every day than cancer-free individuals, regardless of the PA intensity. An inverse dose-response association was also observed between the total PA score and cancer. In addition, the interaction between sex and depressive symptoms and PA significantly affected cancer status.

Our analyses found that cancer survivors were more likely to perform less PA than those who had no cancer diagnoses. This finding may be explained by the fact that some cancer survivors are currently undergoing treatment or recovering from surgery (e.g., adjuvant radiation therapy or chemotherapy), which poses limitations to their physical function and thus prevents or discourages PA. The previous comparison of PA levels between cancer survivors and individuals without cancer remains controversial. The results of the Canadian Community Health Survey (CCHS) are consistent with our results. The CCHS found that respondents with cancer were more likely to be inactive than those who had never had cancer (OR = 1.39, 95% CI = 1.21–1.58 inactive vs. active) (Neil et al., 2014). However, the Lifelines cohort study conducted in a Dutch population contrasted with our study, as the Lifelines study found that cancer survivors were significantly more likely to be physically active than people without a history of cancer (Cortés-Ibáñez et al., 2020). These conflicting findings might be attributed to differences in cancer types, current treatment status, types of treatment, and cancer stages. Researchers in the United States carried out a secondary data analysis to compare PA levels between 31,078 cancer survivors with a history of single-site cancer diagnosis and participants without cancer, and the results demonstrated that higher PA levels were present among prostate cancer survivors, while lower PA levels were present among cervical and endometrial cancer survivors (Kwon et al., 2012). Although evidence has shown that PA throughout treatment is conducive to improving survival and reducing mortality (Demark-Wahnefried et al., 2007; Speck et al., 2010), some cancer treatments can make PA challenging due to their distinct side effects, and even those who participate in PA during treatment may do so at a lower intensity. Future investigations to identify parameters that influence PA among cancer survivors are warranted.

A notable pattern of differences was observed between sexes in the current study, with female cancer patients being less likely to participate in vigorous activity than male cancer patients. A previous study, which found that female cancer survivors were approximately 30% less likely to meet the PA recommendation than male cancer survivors, was in line with our results (LeMasters et al., 2014). This phenomenon may be attributed to the masculinity-femininity theory that a man’s health practices are influenced by his desire to comply with the dominant masculine ideals shaped by cultural norms (e.g., engaging in vigorous PA, smoking, heavy alcohol drinking), while women are more likely to manage their weight through diet adjustment (Wardle et al., 2004; Gough and Conner, 2006). Another theory that may explain the differences in PA levels between sexes is the Health Belief Model, which describes the relationship between an individual’s perception of risk and the corresponding health practices (Cummings et al., 1978). For example, cardiovascular disease is the leading cause of mortality for both men and women in the United States; however, it has been regarded as a “man’s disease,” and only half of women recognize it as a leading cause of death among women (Mosca et al., 2010). Therefore, the lack of perception of risk for developing cardiovascular disease may lead to less PA among females than males. In addition, female cancer survivors seemed to be more vulnerable to mental health declines and sleep disturbance than male cancer survivors, which may also lower their PA levels (LeMasters et al., 2013).

The association between PA level and cancer was more prominent in depressed participants, and cancer survivors with depressive symptoms were less likely to engage in moderate- and vigorous-intensity PA. Researchers found that a depressed mood is negatively related to PA levels among cancer survivors, which may explain the differences observed in depressive status (Galiano-Castillo et al., 2014). We also found an inverse dose-response relationship between the total PA score and cancer status, which might provide new insight into the amount of PA needed for cancer survivors. The finding of significant associations for vigorous activity for more than 10 min weekly but not for light or moderate activity, along with significant relationships for activity for more than 30 min despite PA intensity, suggests that there is a minimum threshold of PA intensity and duration for cancer survivors.

### Strengths and Limitations

The strengths of this study are as follows. First, relatively recent data from a representative middle-aged and older Chinese sample were utilized in this analysis, which improves the generalizability and validity of our findings. Unlike previous studies involving a single intensity of PA, the current study examined different PA intensities and durations in cancer survivors and those without cancer. Moreover, a variety of covariates, including demographic characteristics, health-related lifestyle factors and chronic comorbidities that previous studies failed to fully adjust for, were included in our analysis. Adjustments for these confounders may have resulted in more reliable conclusions since cancer survivors can differ from cancer-free individuals in diverse factors, such as age, sex, BMI, and education levels (Naik et al., 2016).

However, this study also has several limitations. First, the study relied on self-reported PA levels, which may overestimate or underestimate the amount of time spent performing PA compared with objectively measured PA levels. Previous findings suggested that measurement methods may have a significant influence on observed PA levels, and participants may answer questions according to what they think is socially desirable (Prince et al., 2008; Troiano et al., 2008). Nevertheless, a previous study suggested that such bias could potentially be overcome with a large sample size (e.g., CHARLS) (Celis-Morales et al., 2012). Detailed cancer-related medical information, such as cancer diagnosis time, treatment status, and treatment methods, was unavailable in the CHARLS data, which may have limited the interpretation of our results. We only described the sites of cancer because the sample size for each specific cancer was relatively small, which restricted further analyses, as no firm results could be generated. In addition, asking cancer survivors about the levels of PA a few years before their diagnosis was not reliable because of recall bias; thus, PA levels before diagnosis were not measured. Finally, due to the cross-sectional design, it is difficult to know which came first, the physically inactive behavior or the cancer occurrence. The time sequence between PA and cancer was not ascertained; thus, the causality associations between PA and cancer could not be established. Further prospective surveys are needed.

### Implications

Participating in PA is an inexpensive and non-pharmacologic intervention for cancer patients with numerous benefits, such as potential improvements in survival, physical function, quality of life and mortality (Li et al., 2016). The current study found that cancer survivors were less likely to be physically active than individuals without cancer, especially females and those with depressive symptoms.

This may support the introduction of PA into the management of cancer survivors to some extent. Studies have suggested that the period after cancer diagnosis is a “teachable moment,” in which patients are likely to change their lifestyles to improve health outcomes (Blanchard et al., 2003). Therefore, in clinical practice, health care practitioners, especially nurses, can play an important role in monitoring PA levels among cancer survivors. As nurses are close to cancer survivors, they can inform survivors of the beneficial effects of PA and provide valuable advice on recommended PA levels. Moreover, in circumstances where accelerometers are available, nurses and survivors can monitor both PA duration and intensity objectively.

Currently, China houses the world’s largest population with 1.4 billion people, and it is transforming into an aging country (Zeng, 2012; Fang et al., 2015). It is expected that there will be up to 400 million people aged more than 65 years in China by 2030, accounting for 26.9% of the total population (Zeng, 2012). With the rapid growth of the elderly population, the incidence of cancer is also growing rapidly, and significant advancements in medical care and treatment methods have been made (Yang et al., 2008; Chen et al., 2016). Thus, the cancer population is increasing in a parallel manner, and China is encountering formidable healthcare challenges brought about by the problem of cancer survivors. Healthy lifestyle behaviors, including PA, have an important role in both preventing cancer and improving survival and quality of life among cancer survivors and are now receiving attention. Providing training for nurses and establishing multidisciplinary teams (e.g., oncologists, nutritionists, physiotherapists, and nurses) should be considered.

## Conclusion

We found significantly lower PA levels among cancer survivors than among cancer-free individuals after adjustment for demographic characteristics, health-related behaviors, and chronic physical comorbidities. Furthermore, female and depressed cancer survivors were less likely to be physically active and should be given more attention. Our findings demonstrate the need for more prospective studies investigating different PA levels in Chinese cancer survivors.

## Data Availability Statement

The datasets presented in this study can be found in online repositories. The names of the repository/repositories and accession number(s) can be found below: http://charls.pku.edu.cn/index.html.

## Ethics Statement

The studies involving human participants were reviewed and approved by the Institutional Review Board at Peking University. The patients/participants provided their written informed consent to participate in this study.

## Author Contributions

CZ was in charge of the conception and design of the study, as well as the writing of original draft. ZL was responsible for the review and editing of the manuscript. JW and YC reviewed, edited, and supervised this study. All authors read and approved the final manuscript.

## Conflict of Interest

The authors declare that the research was conducted in the absence of any commercial or financial relationships that could be construed as a potential conflict of interest.

## Publisher’s Note

All claims expressed in this article are solely those of the authors and do not necessarily represent those of their affiliated organizations, or those of the publisher, the editors and the reviewers. Any product that may be evaluated in this article, or claim that may be made by its manufacturer, is not guaranteed or endorsed by the publisher.
