# Supplementary material for: Physical Activity and Cancer Status Among Middle-Aged and Older Chinese: A Population-Based, Cross-Sectional Study
Source: Front Physiol. 2022 Jan 27;12:812290. doi: 10.3389/fphys.2021.812290 (PMC8830231; doi:10.3389/fphys.2021.812290)
Supplement: Supplementary file 1 [file Table_1.docx]

**Physical activity and cancer status among middle-aged and older Chinese: a population-based, cross-sectional study**

**Supplementary material**

| **No.** | **Contents** | **Page No.** |
| --- | --- | --- |
| 1 | Supplementary Tables | 2-16 |
| 2 | Supplementary Figure | 17 |
| 3 | The Modified IPAQ-SF | 18 |
| 4 | R code | 19-32 |
| 5 | SPSS syntax | 33-34 |

**Table S1. Physical activity levels between cancer survivors and cancer-free participants**

| Variables | Cancer survivors | Non-cancer | *p value* |
| --- | --- | --- | --- |
| Taking part in activity more than 10 minutes a week |  |  | <.001 |
| Inactive | 51(12.3) | 1286(8.8) |  |
| Light | 149(35.8) | 4159(28.5) |  |
| Moderate | 122(29.3) | 4189(28.7) |  |
| Vigorous | 94(22.6) | 4940(33.9) |  |
| Time usually spent doing activity ≥30min one day |  |  | <.001 |
| Inactive | 93(22.4) | 2131(14.6) |  |
| Light | 132(31.7) | 4017(27.6) |  |
| Moderate | 101(24.3) | 3636(24.9) |  |
| Vigorous | 90(21.6) | 4790(32.9) |  |
| Time usually spent doing activity ≥2 hours one day |  |  | <.001 |
| Inactive | 246(59.1) | 6330(43.4) |  |
| Light | 62(14.9) | 2260(15.5) |  |
| Moderate | 47(11.3) | 2087(14.3) |  |
| Vigorous | 61(14.7) | 3897(26.7) |  |
| Time usually spent doing activity≥4 hours one day |  |  | <.001 |
| Inactive | 321(77.2) | 9678(66.4) |  |
| Light | 22(5.3) | 1015（7.0） |  |
| Moderate | 28(6.7) | 1129(7.7） |  |
| Vigorous | 45(10.8) | 2752(18.9) |  |
| Total PA |  |  | <.001 |
| Q1 | 110(26.4) | 2759(18.9) |  |
| Q2 | 142(34.1) | 4484(30.8) |  |
| Q3 | 94(22.6) | 3653(25.1) |  |
| Q4 | 70(16.8) | 3678(25.2) |  |

Abbreviations: PA=physical activity. Chi square test were used.

**Table S2. Associations between self-reported cancer and other factors**

| Variables | OR(95%CI) | *P-value* |
| --- | --- | --- |
| Age, year |  | .397 |
| <65 | 1 |  |
| ≥65 | 1.10(0.89,1.36) |  |
| Gender, n (%) |  | .003 |
| male | 1 |  |
| female | 1.63(1.18,2.24) |  |
| Education, n (%) |  | .417 |
| below high school | 1 |  |
| high school and above | 1.15(0.83,1.57) |  |
| Marital status, n (%) |  | .368 |
| married | 1 |  |
| separated/divorce/widowed/never married | 0.88(0.66,1.17) |  |
| Place of residence, n (%) |  | .076 |
| rural | 1 |  |
| urban | 1.23(0.98,1.54) |  |
| BMI, kg/m_2_, n (%) |  | .075 |
| <27.9(not obesity) | 1 |  |
| ≥28.0(obesity) | 1.27(0.98,1.64) |  |
| Smoking status, n (%) |  | .001 |
| never | 1 |  |
| former smoker | 1.60(1.13,2.27) | .007 |
| current smoker | 0.89(0.62,1.26) | .497 |
| Drinking frequency, n (%) |  | .232 |
| never | 1 | .351 |
| ≤1/month | 1.19(0.83,1.70) | .223 |
| > 1/ month | 0.83(0.62,1.11) |  |
| Sleep duration, hours, n (%) |  | .002 |
| 7-8 | 1 |  |
| <7 | 1.04(0.79,1.37) | .760 |
| ≥8 | 0.66(0.47,0.92) | .013 |
| Depressive symptoms, n (%) |  | .001 |
| no | 1 |  |
| yes | 1.46(1.19,1.79) |  |
| Comorbidities, n (%) |  | .005 |
| 0 | 1 |  |
| 1 | 1.42(1.13,1.79) | .003 |
| ≥2 | 1.41(1.09,1.84) | .010 |

Abbreviations: BMI=body mass index, OR=odds ratio, CI=confidence interval.

**Table S3. Association between self-reported cancer and physical activity, stratified by age**

| Variables | <65 | ≥65 | *P for heterogeneity* |
| --- | --- | --- | --- |
|  | OR(95%CI) | OR(95%CI) |  |
| Taking part in activity more than 10 minutes a week |  |  | .376 |
| Inactive | 1 | 1 |  |
| Light | 1.42(0. 80,2.49) | 0.73(0.48,1.12) |  |
| Moderate | 0.97(0.55,1.71) | 0.66(0.42,1.04) |  |
| Vigorous | 0.81(0.45,1.43) | 0.45(0.27,0.76) |  |
| Time usually spent doing activity ≥30min one day |  |  | .595 |
| Inactive | 1 | 1 |  |
| Light | 0.96(0.63,1.46) | 0.66(0.46,0.96) |  |
| Moderate | 0.75(0.49,1.16) | 0.55(0.36,0.85) |  |
| Vigorous | 0.64(0.41,0.97) | 0.39(0.24,0.64) |  |
| Time usually spent doing activity ≥2 hours one day |  |  | .340 |
| Inactive | 1 | 1 |  |
| Light | 0.82(0.56,1.21) | 0.63(0.41,0.98) |  |
| Moderate | 0.75(0.51,1.09) | 0.38(0.20,0.71) |  |
| Vigorous | 0.56(0.39,0.81) | 0.34(0.20,0.59) |  |
| Time usually spent doing activity≥4 hours one day |  |  | .389 |
| Inactive | 1 | 1 |  |
| Light | 0.62(0.34,1.12) | 0.84(0.44,1.61) |  |
| Moderate | 0.93(0.59,1.47) | 0.58(0.25,1.33) |  |
| Vigorous | 0.69(0.48,1.01) | 0.40(0.20,0.81) |  |
| Total PA score |  |  | .965 |
| Q1 | 1 | 1 |  |
| Q2 | 0.81(0.56,1.17) | 0.83(0.58,1.18) |  |
| Q3 | 0.68(0.46,0.99) | 0.64(0.41,0.99) |  |
| Q4 | 0.60(0.40,0.89) | 0.50(0.29,0.86) |  |
| *P-trend* | .008 | .005 |  |

Abbreviations: PA=physical activity, OR=odds ratio, CI=confidence interval.

**Table S4. Association between self-reported cancer and physical activity, stratified by gender**

| Variables | Male | Female | *P for heterogeneity* |
| --- | --- | --- | --- |
|  | OR(95%CI) | OR(95%CI) |  |
| Taking part in activity more than 10 minutes a week |  |  | .269 |
| Inactive | 1 | 1 |  |
| Light | 0.87(0.51,1.49) | 1.00(0.66,1.51) |  |
| Moderate | 0.50(0.27,0.94) | 0.85(0.56,1.29) |  |
| Vigorous | 0.53(0.30,0.95) | 0.57(0.36,0.91) |  |
| Time usually spent doing activity ≥30min one day |  |  | .139 |
| Inactive | 1 | 1 |  |
| Light | 0.74(0.48,1.14) | 0.82(0.57,1.16) |  |
| Moderate | 0.40(0.23,0.71) | 0.76(0.53,1.08) |  |
| Vigorous | 0.46(0.29,0.75) | 0.52(0.35,0.78) |  |
| Time usually spent doing activity ≥2 hours one day |  |  | .220 |
| Inactive | 1 | 1 |  |
| Light | 0.71(0.44,1.16) | 0.73(0.51,1.04) |  |
| Moderate | 0.33(0.16,0.69) | 0.72(0.50,1.03) |  |
| Vigorous | 0.43(0.27,0.69) | 0.49(0.36,0.71) |  |
| Time usually spent doing activity≥4 hours one day |  |  | .477 |
| Inactive | 1 | 1 |  |
| Light | 0.81(0.39,1.68) | 0.65(0.37,1.12) |  |
| Moderate | 0.61(0.28,1.32) | 0.91(0.58,1.45) |  |
| Vigorous | 0.49(0.28,0.84) | 0.67(0.45,1.01) |  |
| Total PA score |  |  | .840 |
| Q1 | 1 | 1 |  |
| Q2 | 0.79(0.51,1.20) | 0.85(0.61,1.17) |  |
| Q3 | 0.63(0.39,1.04) | 0.67(0.47,0.95) |  |
| Q4 | 0.49(0.29,0.83) | 0.60(0.41,0.89) |  |
| *P-trend* | .005 | .004 |  |

Abbreviations: PA=physical activity, OR=odds ratio, CI=confidence interval.

**Table S5. Association between self-reported cancer and physical activity, stratified by place of residence**

| Variables | Rural | Urban | *P for heterogeneity* |
| --- | --- | --- | --- |
|  | OR(95%CI) | OR(95%CI) |  |
| Taking part in activity more than 10 minutes a week |  |  | .575 |
| Inactive | 1 | 1 |  |
| Light | 0.87(0.59,1.27) | 1.24(0.63,2.43) |  |
| Moderate | 0.73(0.49,1.08) | 0.83(0.42,1.66) |  |
| Vigorous | 0.56(0.38,0.84) | 0.53(0.23,1.22) |  |
| Time usually spent doing activity ≥30min one day |  |  | .760 |
| Inactive | 1 | 1 |  |
| Light | 0.75(0.54,1.05) | 0.86(0.52,1.40) |  |
| Moderate | 0.65(0.46,0.93) | 0.63(0.37,1.06) |  |
| Vigorous | 0.52(0.37,0.73) | 0.40(0.20,0.82) |  |
| Time usually spent doing activity ≥2 hours one day |  |  | .055 |
| Inactive | 1 | 1 |  |
| Light | 0.62(0.44,0.89) | 1.00(0.63,1.59) |  |
| Moderate | 0.58(0.40,0.85) | 0.64(0.36,1.14) |  |
| Vigorous | 0.50(0.36,0.70) | 0.14(0.03,0.56) |  |
| Time usually spent doing activity≥4 hours one day |  |  | .509 |
| Inactive | 1 | 1 |  |
| Light | 0.73(0.45,1.20) | 0.62(0.23,1.72) |  |
| Moderate | 0.86(0.55,1.33) | 0.69(0.27,1.72) |  |
| Vigorous | 0.63(0.45,0.88) | 0.26(0.06,1.06) |  |
| Total PA score |  |  | .458 |
| Q1 | 1 | 1 |  |
| Q2 | 0.77(0.56,1.05) | 0.94(0.60,1.48) |  |
| Q3 | 0.66(0.47,0.93) | 0.68(0.41,1.13) |  |
| Q4 | 0.59(0.41,0.83) | 0.37(0.16,0.85) |  |
| *P-trend* | .002 | .009 |  |

Abbreviations: PA=physical activity, OR=odds ratio, CI=confidence interval.

**Table S6. Association between self-reported cancer and physical activity, stratified by marital status**

| Variables | Married | Separated/widowed/divorced/never married | *P for heterogeneity* |
| --- | --- | --- | --- |
|  | OR(95%CI) | OR(95%CI) |  |
| Taking part in activity more than 10 minutes a week |  |  | .629 |
| Inactive | 1 | 1 |  |
| Light | 0.93(0.65,1.35) | 1.04(0.50,2.15) |  |
| Moderate | 0.77(0.53,1.12) | 0.59(0.26,1.38) |  |
| Vigorous | 0.54(0.37,0.80) | 0.73(0.30,1.77) |  |
| Time usually spent doing activity ≥30min one day |  |  | .859 |
| Inactive | 1 | 1 |  |
| Light | 0.77(0.57,1.04) | 0.86(0.46,1.63) |  |
| Moderate | 0.66(0.48,0.91) | 0.52(0.24,1.13) |  |
| Vigorous | 0.49(0.36,0.69) | 0.52(0.23,1.22) |  |
| Time usually spent doing activity ≥2 hours one day |  |  | .871 |
| Inactive | 1 | 1 |  |
| Light | 0.69(0.50,0.95) | 0.93(0.49,1.77) |  |
| Moderate | 0.60(0.43,0.85) | 0.55(0.21,1.42) |  |
| Vigorous | 0.46(0.34,0.63) | 0.42(0.16,1.10) |  |
| Time usually spent doing activity≥4 hours one day |  |  | .718 |
| Inactive | 1 | 1 |  |
| Light | 0.84(0.56,1.27) | 0.51(0.12,2.14) |  |
| Moderate | 0.56(0.40,0.80) | 0.79(0.30,2.05) |  |
| Vigorous | 0.75(0.47,1.19) | 0.44(0.11,1.82) |  |
| Total PA score |  |  | .248 |
| Q1 | 1 | 1 |  |
| Q2 | 0.76(0.57,1.01) | 1.22(0.66,2.27) |  |
| Q3 | 0.67(0.49,0.89) | 0.64(0.28,1.46) |  |
| Q4 | 0.51(0.36,0.71) | 1.03(0.44,2.42) |  |
| *P-trend* | <.001 | .582 |  |

Abbreviations: PA=physical activity, OR=odds ratio, CI=confidence interval.

**Table S7. Association between self-reported cancer and physical activity, stratified by education level**

| Variables | Below high school | High school and above | *P for heterogeneity* |
| --- | --- | --- | --- |
|  | OR(95%CI) | OR(95%CI) |  |
| Taking part in activity more than 10 minutes a week |  |  | .732 |
| Inactive | 1 | 1 |  |
| Light | 0.95(0.67,1.34) | 0.88(0.28,2.73) |  |
| Moderate | 0.79(0.55,1.12) | 0.45(0.14,1.47) |  |
| Vigorous | 0.56(0.38,0.81) | 0.55(0.16,1.90) |  |
| Time usually spent doing activity ≥30min one day |  |  | .557 |
| Inactive | 1 | 1 |  |
| Light | 0.84(0.63,1.13) | 0.44(0.20,0.97) |  |
| Moderate | 0.68(0.50,0.93) | 0.39(0.17,0.89) |  |
| Vigorous | 0.50(0.36,0.69) | 0.46(0.19,1.16) |  |
| Time usually spent doing activity ≥2 hours one day |  |  | .222 |
| Inactive | 1 | 1 |  |
| Light | 0.77(0.57,1.03) | 0.45(0.17,1.17) |  |
| Moderate | 0.61(0.44,0.86) | 0.47(0.17,1.37) |  |
| Vigorous | 0.42(0.31,0.58) | 1.22(0.50,2.99) |  |
| Time usually spent doing activity≥4 hours one day |  |  | .204 |
| Inactive | 1 | 1 |  |
| Light | 0.72(0.46,1.12） | 0.47(0.06,3.51) |  |
| Moderate | 0.82(0.54,1.23) | 0.71(0.16,3.04) |  |
| Vigorous | 0.52(0.37,0.73) | 2.10(0.80,5.53) |  |
| Total PA score |  |  | .683 |
| Q1 | 1 | 1 |  |
| Q2 | 0.85(0.65,1.12) | 0.63(0.30,1.31) |  |
| Q3 | 0.70(0.52,0.95) | 0.40(0.17,0.92) |  |
| Q4 | 0.55(0.39,0.76) | 0.62(0.24,1.59) |  |
| *P-trend* | <.001 | .11 |  |

Abbreviations: PA=physical activity, OR=odds ratio, CI=confidence interval.

**Table S8. Association between self-reported cancer and physical activity, stratified by BMI**

| Variables | No (<28.0kg/m^2^) | Obesity (≥28.0kg/m^2^) | *P for heterogeneity* |
| --- | --- | --- | --- |
|  | OR(95%CI) | OR(95%CI) |  |
| Taking part in activity more than 10 minutes a week |  |  | .784 |
| Inactive | 1 | 1 |  |
| Light | 0.90（0.63,1.29) | 1.28(0.59,2.78) |  |
| Moderate | 0.71(0.49,1.03) | 0.93(0.41,2.08) |  |
| Vigorous | 0.54(0.36,0.79) | 0.68(0.27,1.71) |  |
| Time usually spent doing activity ≥30min one day |  |  | .885 |
| Inactive | 1 | 1 |  |
| Light | 0.81(0.60,1.10) | 0.72(0.39,1.32) |  |
| Moderate | 0.67(0.48,0.93) | 0.54(0.28,1.05) |  |
| Vigorous | 0.51(0.36,0.71) | 0.47(0.22,1.02) |  |
| Time usually spent doing activity ≥2 hours one day |  |  | .524 |
| Inactive | 1 | 1 |  |
| Light | 0.68(0.49,0.93) | 0.96(0.52,1.79) |  |
| Moderate | 0.56(0.40,0.80) | 0.76(0.34,1.59) |  |
| Vigorous | 0.46(0.34,0.63) | 0.40(0.17,0.98) |  |
| Time usually spent doing activity≥4 hours one day |  |  | .571 |
| Inactive | 1 | 1 |  |
| Light | 0.62(0.38,1.02) | 1.20(0.47,3.11) |  |
| Moderate | 0.70(0.45,1.10) | 1.45(0.60,3.50) |  |
| Vigorous | 0.54(0.38,0.77) | 0.80(0.33,1.95) |  |
| Total PA score |  |  | .438 |
| Q1 | 1 | 1 |  |
| Q2 | 0.86(0.64,1.14) | 0.70(0.39,1.25) |  |
| Q3 | 0.72(0.53,0.98) | 0.44(0.22,0.89) |  |
| Q4 | 0.53(0.38,0.75) | 0.74(0.35,1.56) |  |
| *P-trend* | <.001 | .134 |  |

Abbreviations: PA=physical activity, OR=odds ratio, CI=confidence interval.

**Table S9. Association between self-reported cancer and physical activity, stratified by depression**

| Variables | Not depression | Depression | *P for heterogeneity* |
| --- | --- | --- | --- |
|  | OR(95%CI) | OR(95%CI) |  |
| Taking part in activity more than 10 minutes a week |  |  | .268 |
| Inactive | 1 | 1 |  |
| Light | 1.18(0.69,2.02) | 0.82(0.54,1.25) |  |
| Moderate | 0.92(0.53,1.60) | 0.62(0.40,0.98) |  |
| Vigorous | 0.88(0.50,1.54) | 0.38(0.23,0.61) |  |
| Time usually spent doing activity ≥30min one day |  |  | .185 |
| Inactive | 1 | 1 |  |
| Light | 0.89(0.59,1.36) | 0.71(0.49,1.02) |  |
| Moderate | 0.76(0.49,1.18) | 0.54(0.36,0.82) |  |
| Vigorous | 0.75(0.48,1.17) | 0.34(0.22,0.52) |  |
| Time usually spent doing activity ≥2 hours one day |  |  | .579 |
| Inactive | 1 | 1 |  |
| Light | 0.72(0.48,1.08) | 0.73(0.48,1.09) |  |
| Moderate | 0.72(0.47,1.11) | 0.47(0.29,0.76) |  |
| Vigorous | 0.55(0.36,0.84) | 0.39(0.26,0.59) |  |
| Time usually spent doing activity≥4 hours one day |  |  | .553 |
| Inactive | 1 | 1 |  |
| Light | 0.82(0.45,1.49） | 0.60(0.31,1.15) |  |
| Moderate | 0.82(0.46,1.46) | 0.80(0.46,1.37) |  |
| Vigorous | 0.78(0.50,1.22) | 0.45(0.28,0.72) |  |
| Total PA score |  |  | .084 |
| Q1 | 1 | 1 |  |
| Q2 | 1.14(0.77,1.68) | 0.61(0.43,0.88) |  |
| Q3 | 0.86(0.56,1.32） | 0.54(0.36,0.80) |  |
| Q4 | 0.86(0.54,1.37) | 0.38(0.25,0.59) |  |
| *P-trend* | .239 | <.001 |  |

Abbreviations: PA=physical activity, OR=odds ratio, CI=confidence interval.

**Table S10. Association between self-reported cancer and physical activity, stratified by smoking**

| Variables | No | Quit | Current smoker | *P for heterogeneity* |
| --- | --- | --- | --- | --- |
|  | OR(95%CI) | OR(95%CI) | OR(95%CI) |  |
| Taking part in activity more than 10 minutes a week |  | p-inter<0.05 |  | .266 |
| Inactive | 1 | 1 | 1 |  |
| Light | 1.12(0.73,1.73) | 0.57(0.29,1.13) | 1.07(0.47,2.41) |  |
| Moderate | 0.95(0.62,1.47) | 0.32(0.15,0.71) | 0.63(0.25,1.57) |  |
| Vigorous | 0.61(0.38,0.98) | 0.45(0.22,0.93) | 0.59(0.25,1.43) |  |
| Time usually spent doing activity ≥30min one day |  |  |  | .439 |
| Inactive | 1 | 1 | 1 |  |
| Light | 0.81(0.57,1.14) | 0.67(0.37,1.22) | 0.92(0.47,1.80) |  |
| Moderate | 0.75(0.53,1.07) | 0.32(0.15,0.70) | 0.55(0.24,1.26) |  |
| Vigorous | 0.49(0.33,0.72) | 0.49(0.25,0.96) | 0.58(0.28,1.19) |  |
| Time usually spent doing activity ≥2 hours one day |  |  |  | .318 |
| Inactive | 1 | 1 | 1 |  |
| Light | 0.82(0.59,1.15) | 0.50(0.23,1.06) | 0.66(0.31,1.44) |  |
| Moderate | 0.73(0.50,1.04) | 0.42(0.18,1.01) | 0.26(0.08,0.85) |  |
| Vigorous | 0.48(0.33,0.70) | 0.27(0.12,0.61) | 0.60(0.33,1.12) |  |
| Time usually spent doing activity≥4 hours one day |  |  |  | .584 |
| Inactive | 1 | 1 | 1 |  |
| Light | 0.69(0.40,1.18) | 0.59(0.18,1.93) | 0.98(0.35,2.79) |  |
| Moderate | 0.87(0.54,1.39) | 0.59(0.21, 1.68) | 0.77(0.27,2.19) |  |
| Vigorous | 0.65(0.44,0.98) | 0.20(0.06,0.65) | 0.81(0.41,1.59) |  |
| Total PA score |  |  |  | .995 |
| Q1 | 1 | 1 | 1 |  |
| Q2 | 0.86(0.62,1.18) | 0.79(0.43,1.42) | 0.74(0.39,1.42) |  |
| Q3 | 0.64(0.45,0.91) | 0.67(0.34,1.32) | 0.79(0.39,1.61) |  |
| Q4 | 0.57(0.39,0.84) | 0.52(0.24,1.11) | 0.59(0.28,1.23) |  |
| *P-trend* | .001 | .076 | .202 |  |

Abbreviations: PA=physical activity, OR=odds ratio, CI=confidence interval.

**Table S11. Association between self-reported cancer and physical activity, stratified by drinking**

| Variables | No | ≤1/month | >1/month | *P for heterogeneity* |
| --- | --- | --- | --- | --- |
|  | OR(95%CI) | OR(95%CI) | OR(95%CI) |  |
| Taking part in activity more than 10 minutes a week |  |  |  | .536 |
| Inactive | 1 | 1 | 1 |  |
| Light | 0.86(1.23,1.56) | 1.56(0.34,7.28) | 1.47(0.51,4.28) |  |
| Moderate | 0.75(0.51,1.08) | 1.12(0.24,5.24) | 0.63(0.20,1.96) |  |
| Vigorous | 0.50(0.33,0.75) | 0.71(0.14,3.51) | 0.87(0.30,2.55) |  |
| Time usually spent doing activity ≥30min one day |  |  |  | .692 |
| Inactive | 1 | 1 | 1 |  |
| Light | 0.73(0.54,1.01) | 0.68(0.27,1.73) | 1.21(0.57,2.54) |  |
| Moderate | 0.69(0.49,0.95) | 0.41(0.15,1.13) | 0.58(0.25,1.35) |  |
| Vigorous | 0.49(0.34,0.70) | 0.31(0.11,0.90) | 0.67(0.31,1.45) |  |
| Time usually spent doing activity ≥2 hours one day |  |  |  | .443 |
| Inactive | 1 | 1 | 1 |  |
| Light | 0.63(0.45,0.89) | 0.83(0.32,2.13) | 1.16(0.62,2.19) |  |
| Moderate | 0.61(0.42,0.88) | 0.87(0.34,2.27) | 0.38(0.15,0.97) |  |
| Vigorous | 0.45(0.31,0.64) | 0.34(0.11,1.04) | 0.55(0.30,1.03) |  |
| Time usually spent doing activity≥4 hours one day |  |  |  | .971 |
| Inactive | 1 | 1 | 1 |  |
| Light | 0.68(0.41,1.14) | 0.36(0.05,2.70) | 0.95(0.37,2.44) |  |
| Moderate | 0.79(0.50,1.26) | 0.94(0.27,3.25) | 0.75(0.29,1.93) |  |
| Vigorous | 0.60(0.41,0.89) | 0.53(0.18,1.62) | 0.54(0.27,1.10) |  |
| Total PA score |  |  |  | .999 |
| Q1 | 1 | 1 | 1 |  |
| Q2 | 0.80(0.60,1.07) | 1.02(0.39,2.62) | 0.88(0.46,170) |  |
| Q3 | 0.65(0.47,0.90) | 0.82(0.30,2.12） | 0.63(0.30,1.29) |  |
| Q4 | 0.54(0.38,0.79) | 0.57(0.19,1.78) | 0.59(0.29,1.20) |  |
| *P-trend* | <.001 | .271 | .085 |  |

Abbreviations: PA=physical activity, OR=odds ratio, CI=confidence interval.

**Table S12. Association between self-reported cancer and physical activity, stratified by sleep duration**

| Variables | 7-8h | <7h | ≥8h | *P for heterogeneity* |
| --- | --- | --- | --- | --- |
|  | OR(95%CI) | OR(95%CI) | OR(95%CI) |  |
| Taking part in activity more than 10 minutes a week |  |  |  | .006 |
| Inactive | 1 | 1 | 1 |  |
| Light | 2.42(0.84,1.00) | 1.00(0.66,1.51) | 0.48(0.24,0.95) |  |
| Moderate | 0.69(0.22,2.19) | 0.89(0.58,1.36) | 0.56(0.29,1.10) |  |
| Vigorous | 1.37(0.45,4.16) | 0.58(0.37,0.91) | 0.32(0.15,0.69) |  |
| Time usually spent doing activity ≥30min one day |  |  |  | .159 |
| Inactive | 1 | 1 | 1 |  |
| Light | 0.90(0.45,1.80) | 0.87(0.62,1.22) | 0.52(0.28,0.96) |  |
| Moderate | 0.36(0.16,0.84) | 0.79(0.55,1.13) | 0.53(0.28,1.01) |  |
| Vigorous | 0.66(0.31,1.39) | 0.52(0.36,0.76) | 0.36(0.18,0.73) |  |
| Time usually spent doing activity ≥2 hours one day |  |  |  | .427 |
| Inactive | 1 | 1 | 1 |  |
| Light | 1.07(0.57,2.01) | 0.72(0.51,1.03) | 0.49(0.23,1.06) |  |
| Moderate | 0.33(0.12,0.94) | 0.65(0.45,0.96) | 0.65(0.31,1.36) |  |
| Vigorous | 0.65(0.33,1.30) | 0.42(0.29,0.61) | 0.50(0.25,0.99) |  |
| Time usually spent doing activity≥4 hours one day |  |  |  | .657 |
| Inactive | 1 | 1 | 1 |  |
| Light | 1.31(0.58,3.00) | 0.65(0.37,1.15) | 0.32(0.08,1.32) |  |
| Moderate | 0.69(0.24,1.97) | 0.82(0.50,1.33) | 0.89(0.35,2.28) |  |
| Vigorous | 0.69(0.31,1.53) | 0.55(0.37,0.83) | 0.61(0.29,1.33） |  |
| Total PA score |  |  |  | .217 |
| Q1 | 1 | 1 | 1 |  |
| Q2 | 0.81(0.43,1.53) | 0.97(0.70,1.33) | 0.53(0.29,0.94) |  |
| Q3 | 0.47(0.22,0.99) | 0.85(0.60,1.21) | 0.38(0.19,0.75) |  |
| Q4 | 0.66(0.31,1.41) | 0.57(0.38,0.85) | 0.50(0.25,0.99) |  |
| *P-trend* | .107 | .005 | .017 |  |

Abbreviations: PA=physical activity, OR=odds ratio, CI=confidence interval.

**Table S13. Association between self-reported cancer and physical activity, stratified by physical comorbidities**

| Variables | 0 | 1 | ≥2 | *P for heterogeneity* |
| --- | --- | --- | --- | --- |
|  | OR(95%CI) | OR(95%CI) | OR(95%CI) |  |
| Taking part in activity more than 10 minutes a week |  |  |  | .829 |
| Inactive | 1 | 1 | 1 |  |
| Light | 1.02(0.57,1.82) | 1.07(0.61,1.87) | 0.78(0.44,1.40) |  |
| Moderate | 0.76(0.42,1.38) | 0.84(0.47,1.49) | 0.64(0.34,1.18) |  |
| Vigorous | 0.64(0.35,1.15) | 0.49(0.26,0.91) | 0.60(0.31,1.17) |  |
| Time usually spent doing activity ≥30min one day |  |  |  | .810 |
| Inactive | 1 | 1 | 1 |  |
| Light | 0.87(0.54,1.42) | 0.88(0.56,1.37) | 0.59(0.35,0.98) |  |
| Moderate | 0.73(0.44,1.21) | 0.61(0.37,1.00) | 0.60(0.34,1.03) |  |
| Vigorous | 0.58(0.35,0.96) | 0.43(0.26,0.72) | 0.52(0.29,0.93) |  |
| Time usually spent doing activity ≥2 hours one day |  |  |  | .963 |
| Inactive | 1 | 1 | 1 |  |
| Light | 0.71(0.44,1.14) | 0.83(0.53,1.29) | 0.59(0.32,1.08) |  |
| Moderate | 0.61(0.37,1.01) | 0.61(0.36,1.02) | 0.54(0.26,1.09) |  |
| Vigorous | 0.49(0.31,0.76) | 0.40(0.24,0.67) | 0.50(0.27,0.93) |  |
| Time usually spent doing activity≥4 hours one day |  |  |  | .942 |
| Inactive | 1 | 1 | 1 |  |
| Light | 0.75(0.39,1.45) | 0.65(0.31,1.34) | 0.66(0.24,1.82) |  |
| Moderate | 0.64(0.33,1.23) | 0.80(0.42,1.49) | 1.23(0.55,2.74) |  |
| Vigorous | 0.54(0.33,0.90) | 0.54(0.31,0.92) | 0.74(0.37,1.48) |  |
| Total PA score |  |  |  | .574 |
| Q1 | 1 | 1 | 1 |  |
| Q2 | 0.77(0.50,1.20) | 1.04(0.69,1.59) | 0.63(0.39,1.04) |  |
| Q3 | 0.69(0.43,1.10) | 0.67(0.41,1.08) | 0.65(0.38,1.13) |  |
| Q4 | 0.59(0.36,0.96) | 0.49(0.28,0.85) | 0.62(0.33,1.16) |  |
| *P-trend* | .032 | .002 | .117 |  |

Abbreviations: PA=physical activity, OR=odds ratio, CI=confidence interval.

**
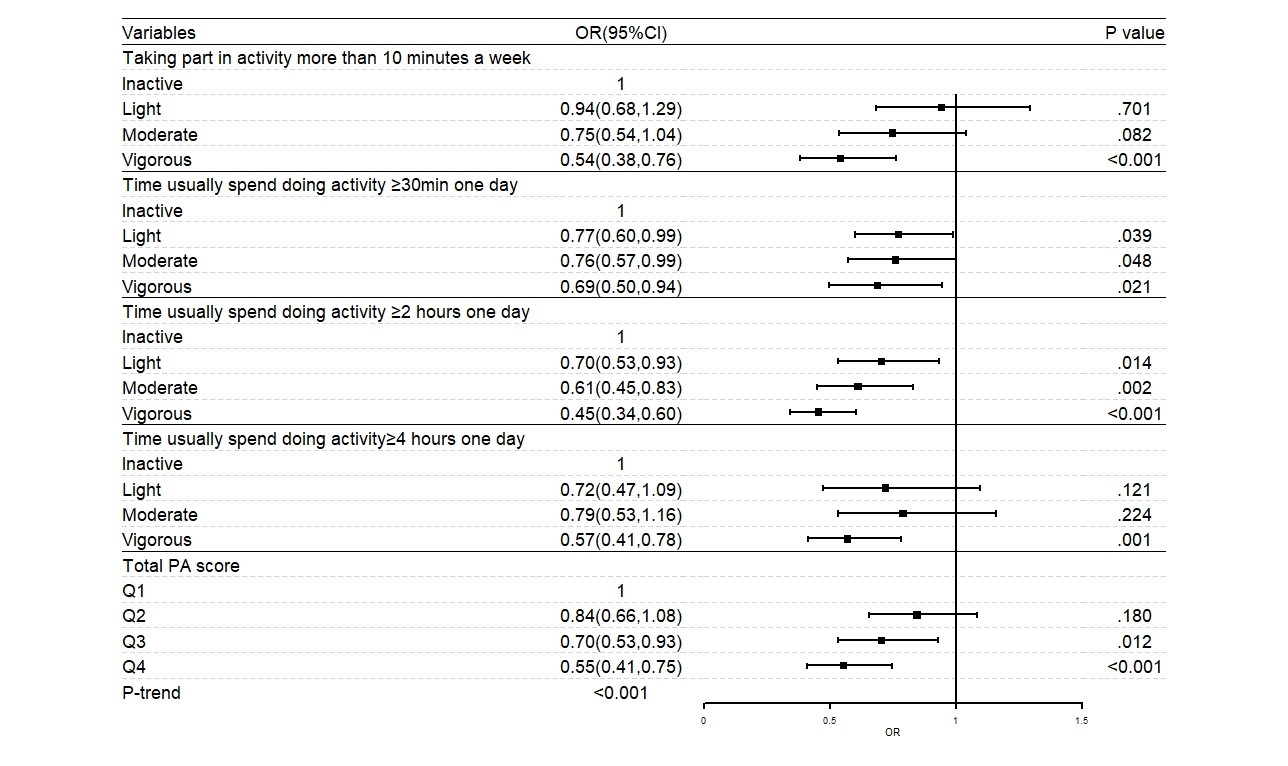
**

**Figure S1.** Sensitivity analyses of association between physical activity levels and cancer survivorship.

Abbreviations: PA=physical activity, OR=odds ratio, CI=confidence interval.

**The modified IPAQ-SF**

Now we would like to ask about the amount of time you spend on different types of physical activities in a usual week.

Now, think about all the vigorous activities requiring hard/high-intensity physical effort that you do in a usual week. Vigorous activities make you breathe much harder than normal and may include heavy lifting, digging, plowing, aerobics, fast bicycling, and cycling with a heavy load. Think only about those physical activities that you did for at least 10 minutes at a time.

1. During a usual week, did you do any vigorous activities for at least 10 minutes continuously?

□ Yes 🡺2

□ No🡺4

2. During a usual week, on how many days did you do vigorous activities for at least 10 minutes?

**└──┘**1…7 days

1. How much time did you usually spend doing vigorous activities on one of those days?

| □< 2 hours | □< 30 minutes  □≥ 30 minutes |
| --- | --- |
| □≥ 2 hours | □< 4 hours  □≥ 4 hours |

Now think about activities which take moderate physical effort that you do in a usual week. Moderate physical activities make you breathe somewhat harder than normal and may include carrying light loads, bicycling at a regular pace, or mopping the floor. Again, think about only those physical activities that you did for at least 10 minutes at a time.

1. During a usual week, did you do any moderate activities for at least 10 minutes continuously?

□ Yes 🡺5

□ No🡺7

1. During a usual week, on how many days did you do moderate activities for at least 10 minutes?

**└──┘**1…7 days

1. How much time did you usually spend doing moderate activities on one of those days?

| □< 2 hours | □< 30 minutes  □≥ 30 minutes |
| --- | --- |
| □≥ 2 hours | □< 4 hours  □≥ 4 hours |

Now think about the time you spend walking in a usual week. This includes at work and at home, walking to travel from place to place, and any other walking that you might do solely for recreation, sport, exercise, or leisure.

1. During a usual week, did you do any walking for at least 10 minutes continuously?

□ Yes 🡺8

□ No

1. During a usual week, on how many days did you do walking for at least 10 minutes?

**└──┘**1…7 days

1. How much time did you usually spend doing walking on one of those days?

| □< 2 hours | □< 30 minutes  □≥ 30 minutes |
| --- | --- |
| □≥ 2 hours | □< 4 hours  □≥ 4 hours |

**R code**

**#Draw Figure 1**

library(haven)

interaction18<-read_sav("D: /interaction.sav")

names(interaction18)

library(plyr)

interaction18<-rename(interaction18,c(HR95CI="OR95%CI",HR="OR"))

View(interaction18)

attach(interaction18)

library(forestplot)

library(grid)

library(magrittr)

library(checkmate)

forestplot(as.matrix(interaction18[,1:3]),OR,LowerCI,UpperCI,graph.pos = 3,zero = 1,graphwidth = unit(200,"mm"),

lineheight = "auto",col = fpColors(all.elements = "black"),boxsize = 0.2,xticks = (c(0,0.5,1,1.5)),

ci.vertices = TRUE,colgap = unit(0,"mm"),hrzl_lines = list("2"=gpar(lwd=1,col="black",lty=1),

"3"=gpar(lwd=1,col="lightgrey",lty=2),

"4"=gpar(lwd=1,col="lightgrey",lty=2),

"5"=gpar(lwd=1,col="lightgrey",lty=2),

"6"=gpar(lwd=1,col="lightgrey",lty=2),

"7"=gpar(lwd=1,col="lightgrey",lty=2),

"8"=gpar(lwd=1,col="lightgrey",lty=2),

"9"=gpar(lwd=1,col="lightgrey",lty=2),

"10"=gpar(lwd=1,col="black",lty=1),

"11"=gpar(lwd=1,col="lightgrey",lty=2),

"12"=gpar(lwd=1,col="lightgrey",lty=2),

"13"=gpar(lwd=1,col="lightgrey",lty=2),

"14"=gpar(lwd=1,col="lightgrey",lty=2),

"15"=gpar(lwd=1,col="lightgrey",lty=2),

"16"=gpar(lwd=1,col="lightgrey",lty=2),

"17"=gpar(lwd=1,col="lightgrey",lty=2),

"18"=gpar(lwd=1,col="lightgrey",lty=2),

"1"=gpar(lwd=1,col="black",lty=1)),

is.summary =c(TRUE,TRUE,rep(FALSE,7),TRUE,rep(FALSE,8)),txt_gp =fpTxtGp(label =gpar(cex=1.5),cex=1.5,xlab=gpar(cex=1.2),ticks = gpar(cex=1)))

detach(interaction18)

**#subgroup analysis**

library(foreign)

library(lattice)

library(survival)

library(Formula)

library(ggplot2)

library(Hmisc)

interaction<-spss.get("D:/2018interaction.sav",use.value.labels=TRUE)

fit1<-glm(cancer2018new~age65+gender+marriage20182+education20182+residence+sleep3+smoking2018+da067+depression18+obesity2+comorbidity3+physi10,family = binomial,data =interaction )

fit2<-glm(cancer2018new~age65+gender+marriage20182+education20182+residence+sleep3+smoking2018+da067+depression18+obesity2+comorbidity3+physi10+physi10*age65,family = binomial,data =interaction )

anova(fit1,fit2,test = "Chisq")

summary(fit1)

exp(coef(fit1))

fit130min<-glm(cancer2018new~age65+gender+marriage20182+education20182+residence+sleep3+smoking2018+da067+depression18+obesity2+comorbidity3+physi30min,family = binomial,data =interaction )

fit230in<-glm(cancer2018new~age65+gender+marriage20182+education20182+residence+sleep3+smoking2018+da067+depression18+obesity2+comorbidity3+physi30min+physi30min*age65,family = binomial,data =interaction )

anova(fit130min,fit230in,test = "Chisq")

#

fit1hour<-glm(cancer2018new~age65+gender+marriage20182+education20182+residence+sleep3+smoking2018+da067+depression18+obesity2+comorbidity3+physical2hour,family = binomial,data =interaction )

fit2hour<-glm(cancer2018new~age65+gender+marriage20182+education20182+residence+sleep3+smoking2018+da067+depression18+obesity2+comorbidity3+physical4hour+physical2hour*age65,family = binomial,data =interaction )

anova(fit1hour,fit2hour,test = "Chisq")

#

fit1hour4<-glm(cancer2018new~age65+gender+marriage20182+education20182+residence+sleep3+smoking2018+da067+depression18+obesity2+comorbidity3+physical4hour,family = binomial,data =interaction )

fit2hour4<-glm(cancer2018new~age65+gender+marriage20182+education20182+residence+sleep3+smoking2018+da067+depression18+obesity2+comorbidity3+physical4hour+physical4hour*age65,family = binomial,data =interaction )

anova(fit1hour4,fit2hour4,test = "Chisq")

#

fit1pa<-glm(cancer2018new~age65+gender+marriage20182+education20182+residence+sleep3+smoking2018+da067+depression18+obesity2+comorbidity3+PA4,family = binomial,data =interaction )

fit2pa<-glm(cancer2018new~age65+gender+marriage20182+education20182+residence+sleep3+smoking2018+da067+depression18+obesity2+comorbidity3+PA4+PA4*age65,family = binomial,data =interaction )

anova(fit1pa,fit2pa,test = "Chisq")

##gender

fit1<-glm(cancer2018new~age65+gender+marriage20182+education20182+residence+sleep3+smoking2018+da067+depression18+obesity2+comorbidity3+physi10,family = binomial,data =interaction )

fit2<-glm(cancer2018new~age65+gender+marriage20182+education20182+residence+sleep3+smoking2018+da067+depression18+obesity2+comorbidity3+physi10+physi10*gender,family = binomial,data =interaction )

anova(fit1,fit2,test = "Chisq")

fit130min<-glm(cancer2018new~age65+gender+marriage20182+education20182+residence+sleep3+smoking2018+da067+depression18+obesity2+comorbidity3+physi30min,family = binomial,data =interaction )

fit230min<-glm(cancer2018new~age65+gender+marriage20182+education20182+residence+sleep3+smoking2018+da067+depression18+obesity2+comorbidity3+physi30min+physi30min*gender,family = binomial,data =interaction)

anova(fit130min,fit230min,test = "Chisq")

fit1hour<-glm(cancer2018new~age65+gender+marriage20182+education20182+residence+sleep3+smoking2018+da067+depression18+obesity2+comorbidity3+physical2hour,family = binomial,data =interaction )

fit2hour<-glm(cancer2018new~age65+gender+marriage20182+education20182+residence+sleep3+smoking2018+da067+depression18+obesity2+comorbidity3+physical2hour+physical2hour*gender,family = binomial,data =interaction)

anova(fit1hour,fit2hour,test = "Chisq")

fit1hour4<-glm(cancer2018new~age65+gender+marriage20182+education20182+residence+sleep3+smoking2018+da067+depression18+obesity2+comorbidity3+physical4hour,family = binomial,data =interaction )

fit2hour4<-glm(cancer2018new~age65+gender+marriage20182+education20182+residence+sleep3+smoking2018+da067+depression18+obesity2+comorbidity3+physical4hour+physical4hour*gender,family = binomial,data =interaction)

anova(fit1hour4,fit2hour4,test = "Chisq")

fit1pa<-glm(cancer2018new~age65+gender+marriage20182+education20182+residence+sleep3+smoking2018+da067+depression18+obesity2+comorbidity3+PA4,family = binomial,data =interaction)

fit2pa<-glm(cancer2018new~age65+gender+marriage20182+education20182+residence+sleep3+smoking2018+da067+depression18+obesity2+comorbidity3+PA4+PA4*gender,family = binomial,data =interaction )

anova(fit1pa,fit2pa,test = "Chisq")

#place of residence

interresidence<-glm(cancer2018new~age65+gender+marriage20182+education20182+residence+sleep3+smoking2018+da067+depression18+obesity2+comorbidity3+physi10+physi10*residence,family = binomial,data =interaction)

fit1<-glm(cancer2018new~age65+gender+marriage20182+education20182+residence+sleep3+smoking2018+da067+depression18+obesity2+comorbidity3+physi10,family = binomial,data =interaction)

anova(fit1,interresidence,test="Chisq")

interresidence1<-glm(cancer2018new~age65+gender+marriage20182+education20182+residence+sleep3+smoking2018+da067+depression18+obesity2+comorbidity3+physi30min+physi30min*residence,family = binomial,data =interaction)

anova(fit130min,interresidence1,test = "Chisq")

interresidence2<-glm(cancer2018new~age65+gender+marriage20182+education20182+residence+sleep3+smoking2018+da067+depression18+obesity2+comorbidity3+physical2hour+physical2hour*residence,family = binomial,data =interaction)

anova(fit1hour,interresidence2,test = "Chisq")

interresidence3<-glm(cancer2018new~age65+gender+marriage20182+education20182+residence+sleep3+smoking2018+da067+depression18+obesity2+comorbidity3+physical4hour+physical4hour*residence,family = binomial,data =interaction)

anova(fit1hour4,interresidence3,test = "Chisq")

interresidence4<-glm(cancer2018new~age65+gender+marriage20182+education20182+residence+sleep3+smoking2018+da067+depression18+obesity2+comorbidity3+PA4+PA4*residence,family = binomial,data =interaction)

anova(fit1pa,interresidence4,test = "Chisq")

#pa interaction with education

interedu<-glm(cancer2018new~age65+gender+marriage20182+education20182+residence+sleep3+smoking2018+da067+depression18+obesity2+comorbidity3+physi10+physi10*education20182,family = binomial,data =interaction)

anova(fit1,interedu,test = "Chisq")

interedu1<-glm(cancer2018new~age65+gender+marriage20182+education20182+residence+sleep3+smoking2018+da067+depression18+obesity2+comorbidity3+physi30min+physi30min*education20182,family = binomial,data =interaction)

anova(fit130min,interedu1,test = "Chisq")

interedu2<-glm(cancer2018new~age65+gender+marriage20182+education20182+residence+sleep3+smoking2018+da067+depression18+obesity2+comorbidity3+physical2hour+physical2hour*education20182,family = binomial,data =interaction)

anova(fit1hour,interedu2,test = "Chisq")

interedu3<-glm(cancer2018new~age65+gender+marriage20182+education20182+residence+sleep3+smoking2018+da067+depression18+obesity2+comorbidity3+physical4hour+physical4hour*education20182,family = binomial,data =interaction)

anova(fit1hour4,interedu3,test = "Chisq")

interedu4<-glm(cancer2018new~age65+gender+marriage20182+education20182+residence+sleep3+smoking2018+da067+depression18+obesity2+comorbidity3+PA4+PA4*education20182,family = binomial,data =interaction)

anova(fit1pa,interedu4,test = "Chisq")

#marital status

intermarital<-glm(cancer2018new~age65+gender+marriage20182+education20182+residence+sleep3+smoking2018+da067+depression18+obesity2+comorbidity3+physi10+physi10*marriage20182,family = binomial,data =interaction)

anova(fit1,intermarital,test = "Chisq")

intermarital1<-glm(cancer2018new~age65+gender+marriage20182+education20182+residence+sleep3+smoking2018+da067+depression18+obesity2+comorbidity3+physi30min+physi30min*marriage20182,family = binomial,data =interaction)

anova(fit130min,intermarital1,test = "Chisq")

intermarital2<-glm(cancer2018new~age65+gender+marriage20182+education20182+residence+sleep3+smoking2018+da067+depression18+obesity2+comorbidity3+physical2hour+physical2hour*marriage20182,family = binomial,data =interaction)

anova(fit1hour,intermarital2,test = "Chisq")

intermarital3<-glm(cancer2018new~age65+gender+marriage20182+education20182+residence+sleep3+smoking2018+da067+depression18+obesity2+comorbidity3+physical4hour+physical4hour*marriage20182,family = binomial,data =interaction)

anova(fit1hour4,intermarital3,test = "Chisq")

intermarital4<-glm(cancer2018new~age65+gender+marriage20182+education20182+residence+sleep3+smoking2018+da067+depression18+obesity2+comorbidity3+PA4+PA4*marriage20182,family = binomial,data =interaction)

anova(fit1pa,intermarital4,test = "Chisq")

#pa interaction with sleep

intersleep<-glm(cancer2018new~age65+gender+marriage20182+education20182+residence+sleep3+smoking2018+da067+depression18+obesity2+comorbidity3+physi10+physi10*sleep3,family = binomial,data =interaction)

anova(fit1,intersleep,test = "Chisq")

intersleep1<-glm(cancer2018new~age65+gender+marriage20182+education20182+residence+sleep3+smoking2018+da067+depression18+obesity2+comorbidity3+physi30min+physi30min*sleep3,family = binomial,data =interaction)

anova(fit130min,intersleep1,test = "Chisq")

intersleep2<-glm(cancer2018new~age65+gender+marriage20182+education20182+residence+sleep3+smoking2018+da067+depression18+obesity2+comorbidity3+physical2hour+physical2hour*sleep3,family = binomial,data =interaction)

anova(fit1hour,intersleep2,test = "Chisq")

intersleep3<-glm(cancer2018new~age65+gender+marriage20182+education20182+residence+sleep3+smoking2018+da067+depression18+obesity2+comorbidity3+physical4hour+physical4hour*sleep3,family = binomial,data =interaction)

anova(fit1hour4,intersleep3,test = "Chisq")

intersleep4<-glm(cancer2018new~age65+gender+marriage20182+education20182+residence+sleep3+smoking2018+da067+depression18+obesity2+comorbidity3+PA4+PA4*sleep3,family = binomial,data =interaction)

anova(fit1pa,intersleep4,test = "Chisq")

# smoking

intersmoking<-glm(cancer2018new~age65+gender+marriage20182+education20182+residence+sleep3+smoking2018+da067+depression18+obesity2+comorbidity3+physi10+physi10*smoking2018,family = binomial,data =interaction)

anova(fit1,intersmoking,test = "Chisq")

intersmoking1<-glm(cancer2018new~age65+gender+marriage20182+education20182+residence+sleep3+smoking2018+da067+depression18+obesity2+comorbidity3+physi30min+physi30min*smoking2018,family = binomial,data =interaction)

anova(fit130min,intersmoking1,test = "Chisq")

intersmoking2<-glm(cancer2018new~age65+gender+marriage20182+education20182+residence+sleep3+smoking2018+da067+depression18+obesity2+comorbidity3+physical2hour+physical2hour*smoking2018,family = binomial,data =interaction)

anova(fit1hour,intersmoking2,test = "Chisq")

intersmoking3<-glm(cancer2018new~age65+gender+marriage20182+education20182+residence+sleep3+smoking2018+da067+depression18+obesity2+comorbidity3+physical4hour+physical4hour*smoking2018,family = binomial,data =interaction)

anova(fit1hour4,intersmoking3,test = "Chisq")

intersmoking4<-glm(cancer2018new~age65+gender+marriage20182+education20182+residence+sleep3+smoking2018+da067+depression18+obesity2+comorbidity3+PA4+PA4*smoking2018,family = binomial,data =interaction)

anova(fit1pa,intersmoking4,test = "Chisq")

#PA interaction with drinking

interdrink<-glm(cancer2018new~age65+gender+marriage20182+education20182+residence+sleep3+smoking2018+da067+depression18+obesity2+comorbidity3+physi10+physi10*da067,family = binomial,data =interaction)

anova(fit1,interdrink,test = "Chisq")

interdrink1<-glm(cancer2018new~age65+gender+marriage20182+education20182+residence+sleep3+smoking2018+da067+depression18+obesity2+comorbidity3+physi30min+physi30min*da067,family = binomial,data =interaction)

anova(fit130min,interdrink1,test = "Chisq")

interdrink2<-glm(cancer2018new~age65+gender+marriage20182+education20182+residence+sleep3+smoking2018+da067+depression18+obesity2+comorbidity3+physical2hour+physical2hour*da067,family = binomial,data =interaction)

anova(fit1hour,interdrink2,test = "Chisq")

interdrink3<-glm(cancer2018new~age65+gender+marriage20182+education20182+residence+sleep3+smoking2018+da067+depression18+obesity2+comorbidity3+physical4hour+physical4hour*da067,family = binomial,data =interaction)

anova(fit1hour4,interdrink3,test = "Chisq")

interdrink4<-glm(cancer2018new~age65+gender+marriage20182+education20182+residence+sleep3+smoking2018+da067+depression18+obesity2+comorbidity3+PA4+PA4*da067,family = binomial,data =interaction)

anova(fit1pa,interdrink4,test = "Chisq")

#PA interation with obesity

interobesity<-glm(cancer2018new~age65+gender+marriage20182+education20182+residence+sleep3+smoking2018+da067+depression18+obesity2+comorbidity3+physi10+physi10*obesity2,family = binomial,data =interaction)

anova(fit1,interobesity,test = "Chisq")

interobesity1<-glm(cancer2018new~age65+gender+marriage20182+education20182+residence+sleep3+smoking2018+da067+depression18+obesity2+comorbidity3+physi30min+physi30min*obesity2,family = binomial,data =interaction)

anova(fit130min,interobesity1,test = "Chisq")

interobesity2<-glm(cancer2018new~age65+gender+marriage20182+education20182+residence+sleep3+smoking2018+da067+depression18+obesity2+comorbidity3+physical2hour+physical2hour*obesity2,family = binomial,data =interaction)

anova(fit1hour,interobesity2,test = "Chisq")

interobesity3<-glm(cancer2018new~age65+gender+marriage20182+education20182+residence+sleep3+smoking2018+da067+depression18+obesity2+comorbidity3+physical4hour+physical4hour*obesity2,family = binomial,data =interaction)

anova(fit1hour4,interobesity3,test = "Chisq")

interobesity4<-glm(cancer2018new~age65+gender+marriage20182+education20182+residence+sleep3+smoking2018+da067+depression18+obesity2+comorbidity3+PA4+PA4*obesity2,family = binomial,data =interaction)

anova(fit1pa,interobesity4,test = "Chisq")

#pa interaction with depression

interdep<-glm(cancer2018new~age65+gender+marriage20182+education20182+residence+sleep3+smoking2018+da067+depression18+obesity2+comorbidity3+physi10+physi10*depression18,family = binomial,data =interaction)

anova(fit1,interdep,test = "Chisq")

interdep1<-glm(cancer2018new~age65+gender+marriage20182+education20182+residence+sleep3+smoking2018+da067+depression18+obesity2+comorbidity3+physi30min+physi30min*depression18,family = binomial,data =interaction)

anova(fit130min,interdep1,test = "Chisq")

interdep2<-glm(cancer2018new~age65+gender+marriage20182+education20182+residence+sleep3+smoking2018+da067+depression18+obesity2+comorbidity3+physical2hour+physical2hour*depression18,family = binomial,data =interaction)

anova(fit1hour,interdep2,test = "Chisq")

interdep3<-glm(cancer2018new~age65+gender+marriage20182+education20182+residence+sleep3+smoking2018+da067+depression18+obesity2+comorbidity3+physical4hour+physical4hour*depression18,family = binomial,data =interaction)

anova(fit1hour4,interdep3,test = "Chisq")

interdep4<-glm(cancer2018new~age65+gender+marriage20182+education20182+residence+sleep3+smoking2018+da067+depression18+obesity2+comorbidity3+PA4+PA4*depression18,family = binomial,data =interaction)

anova(fit1pa,interdep4,test = "Chisq")

#PA interaction with comorbidity

intercomorb<-glm(cancer2018new~age65+gender+marriage20182+education20182+residence+sleep3+smoking2018+da067+depression18+obesity2+comorbidity3+physi10+physi10*comorbidity3,family = binomial,data =interaction)

anova(fit1,intercomorb,test = "Chisq")

intercomorb1<-glm(cancer2018new~age65+gender+marriage20182+education20182+residence+sleep3+smoking2018+da067+depression18+obesity2+comorbidity3+physi30min+physi30min*comorbidity3,family = binomial,data =interaction)

anova(fit130min,intercomorb1,test = "Chisq")

intercomorb2<-glm(cancer2018new~age65+gender+marriage20182+education20182+residence+sleep3+smoking2018+da067+depression18+obesity2+comorbidity3+physical2hour+physical2hour*comorbidity3,family = binomial,data =interaction)

anova(fit1hour,intercomorb2,test = "Chisq")

intercomorb3<-glm(cancer2018new~age65+gender+marriage20182+education20182+residence+sleep3+smoking2018+da067+depression18+obesity2+comorbidity3+physical4hour+physical4hour*comorbidity3,family = binomial,data =interaction)

anova(fit1hour4,intercomorb3,test = "Chisq")

intercomorb4<-glm(cancer2018new~age65+gender+marriage20182+education20182+residence+sleep3+smoking2018+da067+depression18+obesity2+comorbidity3+PA4+PA4*comorbidity3,family = binomial,data =interaction)

anova(fit1pa,intercomorb4,test = "Chisq")

**#interaction analyses**

library(haven)

interaction<-read_sav("E:/ interaction.sav")

View(interaction)

names(interaction)

library(foreign)

library(Hmisc)

library(lattice)

library(survival)

library(Formula)

library(ggplot2)

log_1<-glm(cancer2018new~age65+gender+marriage20182+education20182+residence+sleep3+smoking2018+da067+depression18+obesity2+comorbidity3+physi10,family = binomial,data =interaction )

summary(log_1)

#pa interation with age

interage<-glm(cancer2018new~age65+gender+marriage20182+education20182+residence+sleep3+smoking2018+da067+depression18+obesity2+comorbidity3+physi10+physi10*age65,family = binomial,data =interaction)

summary(interage)

interage1<-glm(cancer2018new~age65+gender+marriage20182+education20182+residence+sleep3+smoking2018+da067+depression18+obesity2+comorbidity3+physi30min+physi10*age65,family = binomial,data =interaction)

summary(interage1)

interage2<-glm(cancer2018new~age65+gender+marriage20182+education20182+residence+sleep3+smoking2018+da067+depression18+obesity2+comorbidity3+physical2hour+physical2hour*age65,family = binomial,data =interaction)

summary(interage2)

interage3<-glm(cancer2018new~age65+gender+marriage20182+education20182+residence+sleep3+smoking2018+da067+depression18+obesity2+comorbidity3+physical4hour+physical4hour*age65,family = binomial,data =interaction)

summary(interage3)

interage4<-glm(cancer2018new~age65+gender+marriage20182+education20182+residence+sleep3+smoking2018+da067+depression18+obesity2+comorbidity3+PA4+PA4*age65,family = binomial,data =interaction)

summary(interage4)

#pa interation with gender

intergender<-glm(cancer2018new~age65+gender+marriage20182+education20182+residence+sleep3+smoking2018+da067+depression18+obesity2+comorbidity3+physi10+physi10*gender,family = binomial,data =interaction)

summary(intergender)#female and physi10(moder) had interaction with

intergender1<-glm(cancer2018new~age65+gender+marriage20182+education20182+residence+sleep3+smoking2018+da067+depression18+obesity2+comorbidity3+physi30min+physi30min*gender,family = binomial,data =interaction)

summary(intergender1)

intergender2<-glm(cancer2018new~age65+gender+marriage20182+education20182+residence+sleep3+smoking2018+da067+depression18+obesity2+comorbidity3+physical2hour+physical2hour*gender,family = binomial,data =interaction)

summary(intergender2)

intergender3<-glm(cancer2018new~age65+gender+marriage20182+education20182+residence+sleep3+smoking2018+da067+depression18+obesity2+comorbidity3+physical4hour+physical4hour*gender,family = binomial,data =interaction)

summary(intergender3)

intergender4<-glm(cancer2018new~age65+gender+marriage20182+education20182+residence+sleep3+smoking2018+da067+depression18+obesity2+comorbidity3+PA4+PA4*gender,family = binomial,data =interaction)

summary(intergender4)

#pa interaction with residence

interresidence<-glm(cancer2018new~age65+gender+marriage20182+education20182+residence+sleep3+smoking2018+da067+depression18+obesity2+comorbidity3+physi10+physi10*residence,family = binomial,data =interaction)

summary(interresidence)

interresidence1<-glm(cancer2018new~age65+gender+marriage20182+education20182+residence+sleep3+smoking2018+da067+depression18+obesity2+comorbidity3+physi30min+physi30min*residence,family = binomial,data =interaction)

summary(interresidence1)

interresidence2<-glm(cancer2018new~age65+gender+marriage20182+education20182+residence+sleep3+smoking2018+da067+depression18+obesity2+comorbidity3+physical2hour+physical2hour*residence,family = binomial,data =interaction)

summary(interresidence2)

interresidence3<-glm(cancer2018new~age65+gender+marriage20182+education20182+residence+sleep3+smoking2018+da067+depression18+obesity2+comorbidity3+physical4hour+physical4hour*residence,family = binomial,data =interaction)

summary(interresidence3)

interresidence4<-glm(cancer2018new~age65+gender+marriage20182+education20182+residence+sleep3+smoking2018+da067+depression18+obesity2+comorbidity3+PA4+PA4*residence,family = binomial,data =interaction)

summary(interresidence4)

#pa interaction with education

interedu<-glm(cancer2018new~age65+gender+marriage20182+education20182+residence+sleep3+smoking2018+da067+depression18+obesity2+comorbidity3+physi10+physi10*education20182,family = binomial,data =interaction)

summary(interedu)

interedu1<-glm(cancer2018new~age65+gender+marriage20182+education20182+residence+sleep3+smoking2018+da067+depression18+obesity2+comorbidity3+physi30min+physi30min*education20182,family = binomial,data =interaction)

summary(interedu1)

interedu2<-glm(cancer2018new~age65+gender+marriage20182+education20182+residence+sleep3+smoking2018+da067+depression18+obesity2+comorbidity3+physical2hour+physical2hour*education20182,family = binomial,data =interaction)

summary(interedu2)

interedu3<-glm(cancer2018new~age65+gender+marriage20182+education20182+residence+sleep3+smoking2018+da067+depression18+obesity2+comorbidity3+physical4hour+physical4hour*education20182,family = binomial,data =interaction)

summary(interedu3)

interedu4<-glm(cancer2018new~age65+gender+marriage20182+education20182+residence+sleep3+smoking2018+da067+depression18+obesity2+comorbidity3+PA4+PA4*education20182,family = binomial,data =interaction)

summary(interedu4)

#pa interaciton with marital status

intermarital<-glm(cancer2018new~age65+gender+marriage20182+education20182+residence+sleep3+smoking2018+da067+depression18+obesity2+comorbidity3+physi10+physi10*marriage20182,family = binomial,data =interaction)

summary(intermarital)

intermarital1<-glm(cancer2018new~age65+gender+marriage20182+education20182+residence+sleep3+smoking2018+da067+depression18+obesity2+comorbidity3+physi30min+physi30min*marriage20182,family = binomial,data =interaction)

summary(intermarital1)

intermarital2<-glm(cancer2018new~age65+gender+marriage20182+education20182+residence+sleep3+smoking2018+da067+depression18+obesity2+comorbidity3+physical2hour+physical2hour*marriage20182,family = binomial,data =interaction)

summary(intermarital2)

intermarital3<-glm(cancer2018new~age65+gender+marriage20182+education20182+residence+sleep3+smoking2018+da067+depression18+obesity2+comorbidity3+physical4hour+physical4hour*marriage20182,family = binomial,data =interaction)

summary(intermarital3)

intermarital4<-glm(cancer2018new~age65+gender+marriage20182+education20182+residence+sleep3+smoking2018+da067+depression18+obesity2+comorbidity3+PA4+PA4*marriage20182,family = binomial,data =interaction)

summary(intermarital4)

#pa interaction with sleep

intersleep<-glm(cancer2018new~age65+gender+marriage20182+education20182+residence+sleep3+smoking2018+da067+depression18+obesity2+comorbidity3+physi10+physi10*sleep3,family = binomial,data =interaction)

summary(intersleep)

intersleep1<-glm(cancer2018new~age65+gender+marriage20182+education20182+residence+sleep3+smoking2018+da067+depression18+obesity2+comorbidity3+physi30min+physi30min*sleep3,family = binomial,data =interaction)

summary(intersleep1)

intersleep2<-glm(cancer2018new~age65+gender+marriage20182+education20182+residence+sleep3+smoking2018+da067+depression18+obesity2+comorbidity3+physical2hour+physical2hour*sleep3,family = binomial,data =interaction)

summary(intersleep2)

intersleep3<-glm(cancer2018new~age65+gender+marriage20182+education20182+residence+sleep3+smoking2018+da067+depression18+obesity2+comorbidity3+physical4hour+physical4hour*sleep3,family = binomial,data =interaction)

summary(intersleep3)

intersleep4<-glm(cancer2018new~age65+gender+marriage20182+education20182+residence+sleep3+smoking2018+da067+depression18+obesity2+comorbidity3+PA4+PA4*sleep3,family = binomial,data =interaction)

summary(intersleep4)

#PA interaction with smoking

intersmoking<-glm(cancer2018new~age65+gender+marriage20182+education20182+residence+sleep3+smoking2018+da067+depression18+obesity2+comorbidity3+physi10+physi10*smoking2018,family = binomial,data =interaction)

summary(intersmoking)

intersmoking1<-glm(cancer2018new~age65+gender+marriage20182+education20182+residence+sleep3+smoking2018+da067+depression18+obesity2+comorbidity3+physi30min+physi30min*smoking2018,family = binomial,data =interaction)

summary(intersmoking1)

intersmoking2<-glm(cancer2018new~age65+gender+marriage20182+education20182+residence+sleep3+smoking2018+da067+depression18+obesity2+comorbidity3+physical2hour+physical2hour*smoking2018,family = binomial,data =interaction)

summary(intersmoking2)

intersmoking3<-glm(cancer2018new~age65+gender+marriage20182+education20182+residence+sleep3+smoking2018+da067+depression18+obesity2+comorbidity3+physical4hour+physical4hour*smoking2018,family = binomial,data =interaction)

summary(intersmoking3)

intersmoking4<-glm(cancer2018new~age65+gender+marriage20182+education20182+residence+sleep3+smoking2018+da067+depression18+obesity2+comorbidity3+PA4+PA4*smoking2018,family = binomial,data =interaction)

summary(intersmoking4)

#PA interaction with drinking

interdrink<-glm(cancer2018new~age65+gender+marriage20182+education20182+residence+sleep3+smoking2018+da067+depression18+obesity2+comorbidity3+physi10+physi10*da067,family = binomial,data =interaction)

summary(interdrink)

interdrink1<-glm(cancer2018new~age65+gender+marriage20182+education20182+residence+sleep3+smoking2018+da067+depression18+obesity2+comorbidity3+physi30min+physi30min*da067,family = binomial,data =interaction)

summary(interdrink1)

interdrink2<-glm(cancer2018new~age65+gender+marriage20182+education20182+residence+sleep3+smoking2018+da067+depression18+obesity2+comorbidity3+physical2hour+physical2hour*da067,family = binomial,data =interaction)

summary(interdrink2)

interdrink3<-glm(cancer2018new~age65+gender+marriage20182+education20182+residence+sleep3+smoking2018+da067+depression18+obesity2+comorbidity3+physical2hour+physical2hour*da067,family = binomial,data =interaction)

summary(interdrink3)

interdrink4<-glm(cancer2018new~age65+gender+marriage20182+education20182+residence+sleep3+smoking2018+da067+depression18+obesity2+comorbidity3+physical4hour+physical4hour*da067,family = binomial,data =interaction)

summary(interdrink4)

#PA interation with obesity

interobesity<-glm(cancer2018new~age65+gender+marriage20182+education20182+residence+sleep3+smoking2018+da067+depression18+obesity2+comorbidity3+physi10+physi10*obesity2,family = binomial,data =interaction)

summary(interobesity)

interobesity1<-glm(cancer2018new~age65+gender+marriage20182+education20182+residence+sleep3+smoking2018+da067+depression18+obesity2+comorbidity3+physi30min+physi30min*obesity2,family = binomial,data =interaction)

summary(interobesity1)

interobesity2<-glm(cancer2018new~age65+gender+marriage20182+education20182+residence+sleep3+smoking2018+da067+depression18+obesity2+comorbidity3+physical2hour+physical2hour*obesity2,family = binomial,data =interaction)

summary(interobesity2)

interobesity3<-glm(cancer2018new~age65+gender+marriage20182+education20182+residence+sleep3+smoking2018+da067+depression18+obesity2+comorbidity3+physical4hour+physical4hour*obesity2,family = binomial,data =interaction)

summary(interobesity3)

interobesity4<-glm(cancer2018new~age65+gender+marriage20182+education20182+residence+sleep3+smoking2018+da067+depression18+obesity2+comorbidity3+PA4+PA4*obesity2,family = binomial,data =interaction)

summary(interobesity4)

#pa interaction with depression

interdep<-glm(cancer2018new~age65+gender+marriage20182+education20182+residence+sleep3+smoking2018+da067+depression18+obesity2+comorbidity3+physi10+physi10*depression18,family = binomial,data =interaction)

summary(interdep)#depression had interaction with physi10, with, vigor

interdep1<-glm(cancer2018new~age65+gender+marriage20182+education20182+residence+sleep3+smoking2018+da067+depression18+obesity2+comorbidity3+physi30min+physi30min*depression18,family = binomial,data =interaction)

summary(interdep1)

interdep2<-glm(cancer2018new~age65+gender+marriage20182+education20182+residence+sleep3+smoking2018+da067+depression18+obesity2+comorbidity3+physical2hour+physical2hour*depression18,family = binomial,data =interaction)

summary(interdep2)

interdep3<-glm(cancer2018new~age65+gender+marriage20182+education20182+residence+sleep3+smoking2018+da067+depression18+obesity2+comorbidity3+physical4hour+physical4hour*depression18,family = binomial,data =interaction)

summary(interdep3)

interdep4<-glm(cancer2018new~age65+gender+marriage20182+education20182+residence+sleep3+smoking2018+da067+depression18+obesity2+comorbidity3+PA4+PA4*depression18,family = binomial,data =interaction)

summary(interdep4)

#PA interaction with comorbidity

intercomorb<-glm(cancer2018new~age65+gender+marriage20182+education20182+residence+sleep3+smoking2018+da067+depression18+obesity2+comorbidity3+physi10+physi10*comorbidity3,family = binomial,data =interaction)

summary(intercomorb)

intercomorb1<-glm(cancer2018new~age65+gender+marriage20182+education20182+residence+sleep3+smoking2018+da067+depression18+obesity2+comorbidity3+physi30min+physi30min*comorbidity3,family = binomial,data =interaction)

summary(intercomorb1)

intercomorb2<-glm(cancer2018new~age65+gender+marriage20182+education20182+residence+sleep3+smoking2018+da067+depression18+obesity2+comorbidity3+physical2hour+physical2hour*comorbidity3,family = binomial,data =interaction)

summary(intercomorb2)

intercomorb3<-glm(cancer2018new~age65+gender+marriage20182+education20182+residence+sleep3+smoking2018+da067+depression18+obesity2+comorbidity3+physical4hour+physical4hour*comorbidity3,family = binomial,data =interaction)

summary(intercomorb3)

intercomorb4<-glm(cancer2018new~age65+gender+marriage20182+education20182+residence+sleep3+smoking2018+da067+depression18+obesity2+comorbidity3+PA4+PA4*comorbidity3,family = binomial,data =interaction)

summary(intercomorb4)

**SPSS syntax**

LOGISTIC REGRESSION VARIABLES cancer2018new

/METHOD=ENTER physi10

/CONTRAST (physi10)=Indicator(1)

/CRITERIA=PIN(.05) POUT(.10) ITERATE(20) CUT(.5).

LOGISTIC REGRESSION VARIABLES cancer2018new

/METHOD=ENTER age gender marriage2 education2 residency2 physi10

/CONTRAST (physi10)=Indicator(1)

/CRITERIA=PIN(.05) POUT(.10) ITERATE(20) CUT(.5).

LOGISTIC REGRESSION VARIABLES cancer2018new

/METHOD=ENTER age gender marriage2 education2 residency2 depression obesity comorbidity

sleep_duraion smoking drinking physi10

/CONTRAST (comorbidity)=Indicator(1)

/CONTRAST (sleep_duraion)=Indicator(1)

/CONTRAST (smoking)=Indicator(1)

/CONTRAST (drinking)=Indicator(1)

/CONTRAST (physi10)=Indicator(1)

/CRITERIA=PIN(.05) POUT(.10) ITERATE(20) CUT(.5).

LOGISTIC REGRESSION VARIABLES cancer2018new

/METHOD=ENTER physi30min

/CONTRAST (physi30min)=Indicator(1)

/CRITERIA=PIN(.05) POUT(.10) ITERATE(20) CUT(.5).

LOGISTIC REGRESSION VARIABLES cancer2018new

/METHOD=ENTER physi30min age gender marriage2 education2 residency2

/CONTRAST (physi30min)=Indicator(1)

/CRITERIA=PIN(.05) POUT(.10) ITERATE(20) CUT(.5).

LOGISTIC REGRESSION VARIABLES cancer2018new

/METHOD=ENTER physi30min age gender marriage2 education2 residency2 sleep_duraion smoking

drinking depression obesity comorbidity

/CONTRAST (physi30min)=Indicator(1)

/CONTRAST (sleep_duraion)=Indicator(1)

/CONTRAST (smoking)=Indicator(1)

/CONTRAST (drinking)=Indicator(1)

/CONTRAST (comorbidity)=Indicator(1)

/CRITERIA=PIN(.05) POUT(.10) ITERATE(20) CUT(.5).

LOGISTIC REGRESSION VARIABLES cancer2018new

/METHOD=ENTER physical2hour

/CONTRAST (physical2hour)=Indicator(1)

/CRITERIA=PIN(.05) POUT(.10) ITERATE(20) CUT(.5).

LOGISTIC REGRESSION VARIABLES cancer2018new

/METHOD=ENTER age gender marriage2 education2 residency2 physical2hour

/CONTRAST (physical2hour)=Indicator(1)

/CRITERIA=PIN(.05) POUT(.10) ITERATE(20) CUT(.5).

LOGISTIC REGRESSION VARIABLES cancer2018new

/METHOD=ENTER age gender marriage2 education2 residency2 sleep_duraion smoking drinking

depression obesity comorbidity physical2hour

/CONTRAST (sleep_duraion)=Indicator(1)

/CONTRAST (smoking)=Indicator(1)

/CONTRAST (drinking)=Indicator(1)

/CONTRAST (comorbidity)=Indicator(1)

/CONTRAST (physical2hour)=Indicator(1)

/CRITERIA=PIN(.05) POUT(.10) ITERATE(20) CUT(.5).

LOGISTIC REGRESSION VARIABLES cancer2018new

/METHOD=ENTER physical4hour

/CONTRAST (physical4hour)=Indicator(1)

/CRITERIA=PIN(.05) POUT(.10) ITERATE(20) CUT(.5).

LOGISTIC REGRESSION VARIABLES cancer2018new

/METHOD=ENTER age gender marriage2 education2 residency2 physical4hour

/CONTRAST (physical4hour)=Indicator(1)

/CRITERIA=PIN(.05) POUT(.10) ITERATE(20) CUT(.5).

LOGISTIC REGRESSION VARIABLES cancer2018new

/METHOD=ENTER age gender marriage2 education2 residency2 sleep_duraion smoking drinking

depression obesity comorbidity physical4hour

/CONTRAST (sleep_duraion)=Indicator(1)

/CONTRAST (smoking)=Indicator(1)

/CONTRAST (drinking)=Indicator(1)

/CONTRAST (comorbidity)=Indicator(1)

/CONTRAST (physical4hour)=Indicator(1)

/CRITERIA=PIN(.05) POUT(.10) ITERATE(20) CUT(.5).

LOGISTIC REGRESSION VARIABLES cancer2018new

/METHOD=ENTER PA4

/CONTRAST (PA4)=Indicator(1)

/CRITERIA=PIN(.05) POUT(.10) ITERATE(20) CUT(.5).

LOGISTIC REGRESSION VARIABLES cancer2018new

/METHOD=ENTER PA4 age gender marriage2 education2 residency2

/CONTRAST (PA4)=Indicator(1)

/CRITERIA=PIN(.05) POUT(.10) ITERATE(20) CUT(.5).

LOGISTIC REGRESSION VARIABLES cancer2018new

/METHOD=ENTER PA4 age gender marriage2 education2 residency2 sleep_duraion smoking drinking

depression obesity comorbidity

/CONTRAST (PA4)=Indicator(1)

/CONTRAST (sleep_duraion)=Indicator(1)

/CONTRAST (smoking)=Indicator(1)

/CONTRAST (drinking)=Indicator(1)

/CONTRAST (comorbidity)=Indicator(1)

/CRITERIA=PIN(.05) POUT(.10) ITERATE(20) CUT(.5).

LOGISTIC REGRESSION VARIABLES cancer2018new

/METHOD=ENTER PA4 age gender marriage2 education2 residency2 sleep_duraion smoking drinking

depression obesity comorbidity

/CONTRAST (sleep_duraion)=Indicator(1)

/CONTRAST (smoking)=Indicator(1)

/CONTRAST (drinking)=Indicator(1)

/CONTRAST (comorbidity)=Indicator(1)

/CRITERIA=PIN(.05) POUT(.10) ITERATE(20) CUT(.5).
